# Probiotic peptidoglycan skeleton enhances vaccine efficacy against MRSA by inducing trained immunity via the TLR2/JAK-STAT3 pathway

**DOI:** 10.3389/fimmu.2025.1606626

**Published:** 2025-07-18

**Authors:** Lingdi Niu, Haoyuan Duan, Jiaqing Wang, Zheng Jia, Hai Li, Junjie Guo, Shuhe Zhang, Ning Liu, Yaxin Miao, Junwei Ge, Fang Wang

**Affiliations:** ^1^ National Key Laboratory for Animal Disease Control and Prevention, Harbin Veterinary Research Institute, Chinese Academy of Agricultural Sciences, Harbin, China; ^2^ Heilongjiang Provincial Key Laboratory of Zoonosis, College of Veterinary Medicine, Northeast Agricultural University, Harbin, China

**Keywords:** peptidoglycan backbone, bacterium-like particles, innate immunity, trained immunity, MRSA

## Abstract

Trained immunity refers to the ability of trained innate immune cells to generate an immune memory that produces rapid, broad-spectrum, and long-lasting protection against heterologous stimuli. Based on the rapid and broad-spectrum protection that the peptidoglycan backbone from lactic acid bacteria, bacterium-like particles (BLPs), offers, we hypothesized that BLPs enhance protection through trained immunity. Here, we found that combining BLP with a vaccine significantly improves protective efficacy against methicillin-resistant *Staphylococcus aureus* (MRSA) infection, accompanied by changes in trained immunity markers. We demonstrate that BLP-induced trained immunity macrophages exhibit increased cytokine secretion and phagocytic activity *in vitro*. In an *in vivo* model, BLP confers protection against *S. aureus* 26003 even without specific antigens. In an *ex vivo* model, BLP induces increased markers of trained immunity. Transcriptome analysis suggests that BLP may induce trained immunity by activating the IL-6-JAK-STAT3 pathway through TLR2 receptor activation, thereby modulating macrophage metabolic reprogramming and function. In summary, our study establishes that BLP induction of trained immunity, along with regulated metabolic reprogramming and macrophage function, may contribute to enhancing vaccine efficacy. Our findings elucidate a novel mechanism for BLP-mediated immune enhancement, critical for the application of BLP as a vaccine vector to construct a vaccine that combines specific immune response with innate immune response.

## 1 Introduction

The clinical application of antibiotics has contributed to the rise of multidrug-resistant (MDR) bacteria, precipitating the current crisis in antibiotic resistance. The morbidity and mortality associated with MDR infections have been increasing in recent years and pose significant economic burdens (1, 2). Bacterial or virus coinfection with methicillin-resistant *Staphylococcus aureus* (MRSA) was an elevated mortality rate risk factor (3). In the 2014 State of the Union address, the importance of investing in research for “vaccines that stay ahead of drug-resistant bacteria” was emphasized (4). Consequently, vaccine development to combat antimicrobial resistance caused by MDR bacteria has become critically important. While traditional vaccine platforms, such as inactivated and live attenuated vaccines, are effective in preventing bacterial infections, they fall short in addressing emerging MDR strains (5). Therefore, there is a pressing need to develop new vaccines and alternative immunotherapies to prevent the emergence of coinfections, MDR infections, and potential epidemics.

The peptidoglycan is a unique and essential structural element in the cell wall of most bacteria. There is a high diversity in the composition, sequence, and biological function of the peptides in the peptidoglycan from different species (6). Bacterium-like particles (BLPs) are hollow particles derived by treating lactic acid bacteria, retaining only the cell wall peptidoglycan skeleton (7–9). To date, BLPs have participated in more than 40 different varieties of bacteria. The BLP surface display platform incorporates BLPs and anchoring proteins, facilitating the presentation of exogenous proteins. BLPs activate DCs via TLR2, enhance T-cell activation, and modulate Th1 and Th2 immunity, thereby eliciting a more potent immune response (9–12). Consequently, BLPs serve as natural adjuvants to augment antigen immunogenicity. Despite their immune-boosting properties, enhancing the immunization efficacy of BLPs remains a critical area for exploration. The mechanism of action of BLPs is still poorly understood, posing a significant hurdle in their development (8). Clarifying the mechanisms underlying the BLP surface display system’s immune-enhancing role will greatly enhance the performance of BLP-based vaccines.

In the past decade, research has identified that certain stimulants can induce innate immune memory, characterized by “memory traits” that lead to heightened reactivity and clearance upon reinfection with the same or different pathogens (13, 14). This phenomenon is termed “trained immunity” (15), where innate immune cells are triggered by identifying pathogen-associated molecular patterns (PAMPs) through cellular pattern recognition receptors (PRRs) (16, 17). This activation initiates metabolic and epigenetic reprogramming, enhancing immune-related processes within immune cells (18–20), thereby providing both specific and nonspecific protection (17, 21). Key agonists identified in trained immunity include β-glucan, a polysaccharide found in fungal cell walls (22, 23). The novel Trained Immunity-based Vaccine (TIbV) offers several advantages over traditional vaccines. Firstly, TIbV harnesses both innate and adaptive immune memories to confer innate cross-protection to heterologous pathogens in addition to specific immunity (24). Secondly, TIbV possesses adjuvant properties that boost adaptive immune responses against both self- and exogenous antigens. Therefore, trained immunity activators have important implications for a response to vaccines or their design (25). Several vaccines, including Bacille Calmette-Guérin (BCG) and COVID-19 vaccines, have demonstrated the ability to induce trained immunity, thereby reducing morbidity and mortality (21, 26–34), with trained innate immune cells exhibiting long-lasting memory responses (35, 36). Identifying new activators or vaccine vectors capable of inducing trained immunity is crucial for designing TIbV vaccines that offer rapid, durable, and broad-spectrum protection upon reinfection (37).

This study aimed to elucidate the mechanism by which BLP enhances vaccine immunity, focusing on its potential to induce trained immunity. To evaluate our hypothesis, we investigated BLP’s ability to induce trained immunity utilizing *in vitro*, *in vivo*, and *ex vivo* models, and elucidated part of its mechanism through transcriptome analysis. Specifically: (i) During the development of a vaccine against MRSA infections, combining the BLP adjuvant with the protein antigen SAMEA (*S. aureus* multi-epitope antigen) significantly enhances vaccine efficacy, resulting in a rapid organismal response to MRSA infection. (ii) BLP was shown to be a potent activator of trained immunity in *in vitro* cellular experiments, inducing alterations in macrophage metabolism and cellular functions. (iii) *In vivo* experiments in mice demonstrated that pretreatment with BLP provides heterologous protection against *S. aureus* infections. (iv) *Ex vivo* models demonstrated that BLP induces trained immunity in peritoneal macrophages, confirming the heterologous protective effect observed *in vivo*. (v) Transcriptome analysis was utilized to dissect the mechanisms by which BLP induces trained immunity. Our study highlights BLP’s ability to induce trained immunity and explores potential mechanisms. These findings are critical for integrating BLP into the design of TIbV vaccines against MDR infections.

## 2 Materials and methods

### 2.1 Strains, animals, and cells

*Levilactobacillus brevis* 23017 (CCTCC AB 2018164) was isolated and identified by our laboratory (38, 39) and cultivated in MRS medium at 37°C. *S. aureus* CMCC26003 was purchased from the China Institute of Veterinary Drug Control (IVDC, Beijing, China). MRSA strain USA300 (ATCC BAA-1717) was provided by Dr. Yanhua Li at Northeast Agricultural University. TSB medium was used to culture *S. aureus* at 37°C. *Escherichia coli* pET28a-GFP/Rosetta, which fluoresces green, is maintained in our laboratory. β-glucan (CAS: 904122-9) was purchased from Sigma-Aldrich (St. Louis, MO, USA).

Female Kunming mice (4–6 weeks old, 18–22 g) were purchased from Changsheng Biological Company (Liaoning, China). All animal experiments in this study were carried out in compliance with the guidelines set by the Laboratory Animal Ethics Committee of Northeast Agricultural University (No. NEAUEC20210326). The animals were free to drink and eat under constant conditions, and the experiments were conducted after the test animals had adapted to the environment.

RAW264.7 cells were purchased from the IVDC. RAW264.7 cells were cultured in Dulbecco’s modified Eagle’s medium (DMEM; Gibco, USA) supplemented with 10% fetal bovine serum (FBS) and 1% penicillin–streptomycin at 37°C.

### 2.2 Bacterium-like particles

As previously mentioned (9), BLPs were prepared through hot acid pretreatment. 23017 strains were suspended in trichloroacetic acid (10%) and boiled for 45 min, then washed three times, and finally stored at −80°C. The particles were counted using a cell counter. The number of BLP particles per milliliter was adjusted according to the colony-forming unit/mL determined in the starting culture (1 U = 2.5 × 10^9^ inactive BLPs) (40).

### 2.3 Preparation of SAMEA

Bioinformatics analysis is becoming more commonly utilized to design multi-epitope vaccines. In our earlier experiments, we predicted and constructed SAMEA (41). Comprehensive physicochemical and structural analyses confirmed SAMEA’s stability, with additional parameters such as its antigenicity, toxicity, and allergenicity aligning with the desired profiles for vaccine efficacy. It includes dominant antigenic epitopes of 10 antigens (clfa, fnbpa, idsb, pnsg, spa, sasx, hla, mntc, seb, and plc), one B cell, one CTL, and one HTL epitope identified through bioinformatic analysis, which were selected from each of the 10 immunodominant antigens (to be published). The SAMEA protein used in this study was an antigen purified using a His-tag protein purification kit (Beyotime, China). After purification, endotoxins were removed using a Protein Endotoxin Removal Kit (Beyotime, China). Subsequently, an endotoxin detection kit was used to measure the LPS content (Beyotime, China).

### 2.4 Vaccination procedure

For immunization, mice were assigned to six groups (*n* = 5). Both the control and infection groups received PBS through intraperitoneal injections (i.p.) on day 0 and day 21; the SAMEA group received SAMEA (50 μg, i.p.) on day 0 and day 21; the SAMEA+ALU group received SAMEA (50 μg) and an equal volume of alum adjuvant on day 0 and day 21 (i.p.); the SAMEA+β-glucan group received SAMEA (50 μg) and β-glucan (500 μg) on day 0 and day 21 (i.p.); the SAMEA+BLP group received SAMEA (50 μg, i.p.) and oral administration with BLP (1 U) on day 0 and day 21. On days 28, 35, and 42, the serum was then extracted from the tail vein for subsequent analysis. On day 42, mice in all groups except the control group were injected with MRSA (5×10^8^ CFU/100 μL) in the tail vein. Mouse body weight and survival were monitored until day 63. Then, the mice were euthanized by cervical dislocation under anesthesia with 4% isoflurane, with relevant samples collected for subsequent analysis.

Experiments were designed according to the methodology of Ferreira et al. (42). For one dose immunization, mice were assigned to six groups (*n* = 5), and both the control and infection groups received PBS on day −3 and day 0 (i.p.); the SAMEA group received PBS on day −3 and SAMEA (50 μg) on day 0 (i.p.); the SAMEA+ALU group received PBS on day −3 and received SAMEA (50 μg) and an equal volume of alum adjuvant on day 0 (i.p.); the SAMEA+β-glucan group received β-glucan (500 μg) on day −3 and day 0 and SAMEA (50 μg) on day 0 (i.p.); the SAMEA+BLP group was oral administrated with BLP (1 U) on day −3 and day 0, and intraperitoneally injected with SAMEA (50 μg) on day 0. On days 7, 14, and 21, the serum was then extracted for subsequent analysis. On day 20, the inflammatory response in mice had completely subsided. On day 21, mice in all groups except the control group were injected with MRSA (5×10^8^ CFU/100 μL) in the tail vein. Mouse body weight and survival were monitored until day 42. Then, the mice were euthanized by cervical dislocation under anesthesia with 4% isoflurane, with relevant samples collected for subsequent analysis.

### 2.5 Trained immunity *in vitro*

The methodology was based on Paris et al. (43); RAW264.7 cells were stimulated with stimulants. The cells were cultured for 2 h to allow them to adhere to the wall. Initial stimulation of RAW264.7 cells was performed by adding PBS, β-glucan (30 µg/mL, a dose that effectively induced trained immunity in previous studies) (44), and BLP (0.01 U/well). Remove stimulus after 24 h. On day 7, cells in each group were stimulated for 24 h with LPS (100 ng/mL) for secondary stimulation. Cells were harvested in Trizol, and culture supernatants were collected in sterile EP tubes for subsequent experiments.

Inhibition experiments were performed according to the method in Paris et al. (43). We used 1 mM of 5’-deoxy-5’-(methylthio) adenosine (MTA) (S880063, Macklin, Shanghai, China) to inhibit the histone methylation. To inhibit glycolysis, macrophages were pre-incubated with 15 mM of 2-deoxy-D-glucose (2-DG, D807272, Macklin, Shanghai, China) for 1 h. Also, 10 μmol/L rapamycin (RAPA, S115842, Aladdin, Shanghai, China) was used to inhibit the mTOR signaling pathway to explore whether this pathway plays a role in trained immunity induced by BLP.

### 2.6 Trained immunity *in vivo*

The methodology was based on Ferreira et al. (42); mice were assigned to four groups (*n* = 5). The control group and infection group were intraperitoneally injected with PBS on day 0 and day 3; the β-glucan group was intraperitoneally injected with β-glucan (500 μg) on day 0 and day 3; the BLP group was orally administered with BLP (1 U) on day 0 and day 3. On day 7, mice in all groups except the control group were intraperitoneally injected with *S. aureus* CMCC26003 (1×10^9^ CFU/200 µL). Normal feeding was observed until day 5, monitored, and recorded for body weight, survival rate, and activity score. Then, the mice were euthanized by cervical dislocation under anesthesia with 4% isoflurane.

Mouse activity was scored for damage based on a simple modification of the methodology of Pasquali et al. (45), with healthy mice scoring 0; mice showing slightly abnormal symptoms of wilting scoring 1–2; mice arching their backs, constricting their abdomens, and being inactive for some time or blowing up their fur scoring 3–5; mice being almost inactive, with ragged coats, bunching up, and becoming moribund scoring 6–9; and mice dying scoring 10.

### 2.7 Trained immunity in *ex vivo*

The methodology was based on Pan et al. (44); mice were assigned to three groups (*n* = 5). The control group was intraperitoneally injected with PBS on day 0 and day 3; the β-glucan group was intraperitoneally injected with β-glucan (500 μg) on day 0 and day 3; the BLP group was orally administered with BLP (1 U) on day 0 and day 3. On day 6, mice were euthanized by cervical dislocation under anesthesia with 4% isoflurane. Peritoneal macrophages were isolated and cultured for 2 h before being stimulated with LPS (100 ng/mL). Peritoneal macrophages were collected at 1, 6, 12, and 24 h post-LPS stimulation for subsequent analysis.

### 2.8 Transcriptome sequencing and analysis

Mouse peritoneal macrophages stimulated with LPS for 6 h were subjected to RNA-seq analysis by NovelBio Bio Pharm Technology Co., Ltd. (Shanghai, China). Total RNA was extracted with Trizol Reagent (Invitrogen, Waltham, MA, USA). Briefly, check the total RNA quality with an Agilent 2200 Bioanalyzer (Agilent Technologies, Santa Clara, CA, USA). RNA with RIN (RNA Integrity Number) greater than 7.0 can be used to construct cDNA libraries. The Agencourt AMPure XP-PCR Purification Beads (Beckman Coulter, Brea, CA, USA) were used to purify and fragment the RNA. The libraries were quality-controlled with an Agilent 2200 and sequenced by DNBSEQ-T7 on a 150-bp paired-end run. The clean reads were then aligned to the Mouse genome (mm10) using Hisat2 (46). HTseq fields (47) were used to obtain gene counts, and the RPKM method was employed to determine gene expression. We applied the DESeq2 algorithm (48) to filter the differentially expressed genes (DEGs). After the significant analysis, *p*-value and FDR analysis were subjected to the following criteria: fold change > 1.5 or <0.667, *p*-value < 0.05, and FDR < 0.05.

We obtained Gene Ontology (GO) annotations from the GO website (http://www.geneontology.org/). Pathway analysis was conducted based on the Kyoto Encyclopedia of Genes and Genomes (KEGG) database. Gene set enrichment analyses (GSEAs) were performed using GSEA software v4.1.0 from the Broad Institute, utilizing the 50 Hallmark gene sets of MSigDB as input (49). Fisher’s exact test was employed to select significant GO categories and pathways, with a significance threshold set at *p* < 0.05 (50).

### 2.9 RNA extraction and RT-qPCR

To detect cytokines, the method described in Shi et al. (39) was followed. Total RNA was extracted (ER101-01, TransGen Biotech, Beijing, China) and reverse transcribed to cDNA (TRT-101, TOYOBO, Shanghai, China), followed by quantification and gene expression analysis (A2250B, HaiGene Biotech Co., Ltd., Harbin, China), with Actin β as the reference gene. The primers were listed in **Supplementary Table S1** as per references (39, 44, 51–56) (Comate Bioscience Co., Ltd., Changchun, Jilin, China). The relative mRNA expression levels were determined using the 2^−ΔΔCT^ method (57).

### 2.10 Measurement of bacterial load

The bacterial load was determined using the method described by Nour et al. (58). The tissues of the mouse in each group were aseptically collected, homogenized in PBS, diluted up to 10^−8^ times the original dilution, and plated out on TSA plates, where the colonies were counted after 18 h at 37°C.

### 2.11 Histopathological detection

Under aseptic conditions, the spleens and kidneys of mice were harvested, fixed in 10% formaldehyde, labeled, and sent to Wuhan Servicebio Technology Co., Ltd. (Wuhan, Hubei, China) for pathological section preparation.

### 2.12 Measurement of antibody response using ELISA

Detection of antibody levels was according to the method of Niu et al. (9); indirect ELISA was employed to assess the levels of SAMEA-specific antibodies IgG, IgG1, and IgG2a. In brief, purified SAMEA antigen (2 µg/mL) was added to a 96-well plate overnight for coating. It was subsequently blocked with 5% skim milk, serum samples were diluted, and diluted antibodies IgG (HRP-conjugated Goat anti-Mouse IgG, AS003, Abclonal, Wuhan, China), IgG1 (HRP-conjugated Goat anti-Mouse IgG1, AS066, Abclonal, Wuhan, China), and IgG2a (HRP-conjugated Goat anti-Mouse IgG2a, AS065, Abclonal, Wuhan, China) were added with 5% skim milk. The reaction used TMB substrate (PR1210, Solarbio, Beijing, China). H_2_SO_4_ (2 M) was used to stop the reaction, which was then measured at 450 nm using a Thermo Fisher spectrophotometer (New York, NY, USA).

### 2.13 Phagocytic activity of RAW 264.7 cells

The BLP was tested to determine whether it induced changes in phagocytosis of Raw264.7 macrophages according to the method in Pan et al. (44), such as RAW264.7 macrophage *in vitro* model treatment of the cells, followed by static incubation of the macrophages overnight, and then co-cultivated with GFP-labeled *E. coli* (*E. coli* strains that fluoresce green) for 1 h. After washing away the unphagocytosed bacteria, a mixture of double antibiotics, gentamicin and ampicillin, was added to eliminate them from the macrophages. While the cells were later stained using DAPI dye (the nucleus fluoresces blue after staining), the phagocytosis ability of each group of Raw264.7 cells was subsequently observed using fluorescence microscopy.

### 2.14 Glucose and lactic acid determination

Lactic acid (LA) and glucose (GLU) levels in the cell supernatants and serum were determined by using the LA and GLU assay kits (Nanjing Jiancheng, Nanjing, China), respectively.

### 2.15 ROS and NO

Nitric oxide (NO) was detected according to the kit (Nanjing Jiancheng, Nanjing, China) instructions; reactive oxygen species (ROS) was detected according to the kit (Nanjing Jiancheng, Nanjing, China) instructions, and its luminescence intensity was detected by a fluorescent enzyme marker.

### 2.16 Statistical analysis

All data were statistically evaluated using GraphPad Prism 9 software. One-way ANOVA was utilized to determine the significance of differences between groups. Survival analysis was conducted with the Kaplan–Meier method, and survival rate differences were examined using the log-rank test. Results are presented as mean ± SD. Statistical significance was defined as *p* < 0.05 for significant differences and *p* < 0.01 for highly significant differences, with **** indicating *p* < 0.0001, ***p < 0.001, **p < 0.01, and *p < 0.05.

## 3 Results

### 3.1 BLP combined with the subunit vaccine enhances protection with rapid recovery and inflammation resolution in mice challenged by MRSA

**Figure 1:**
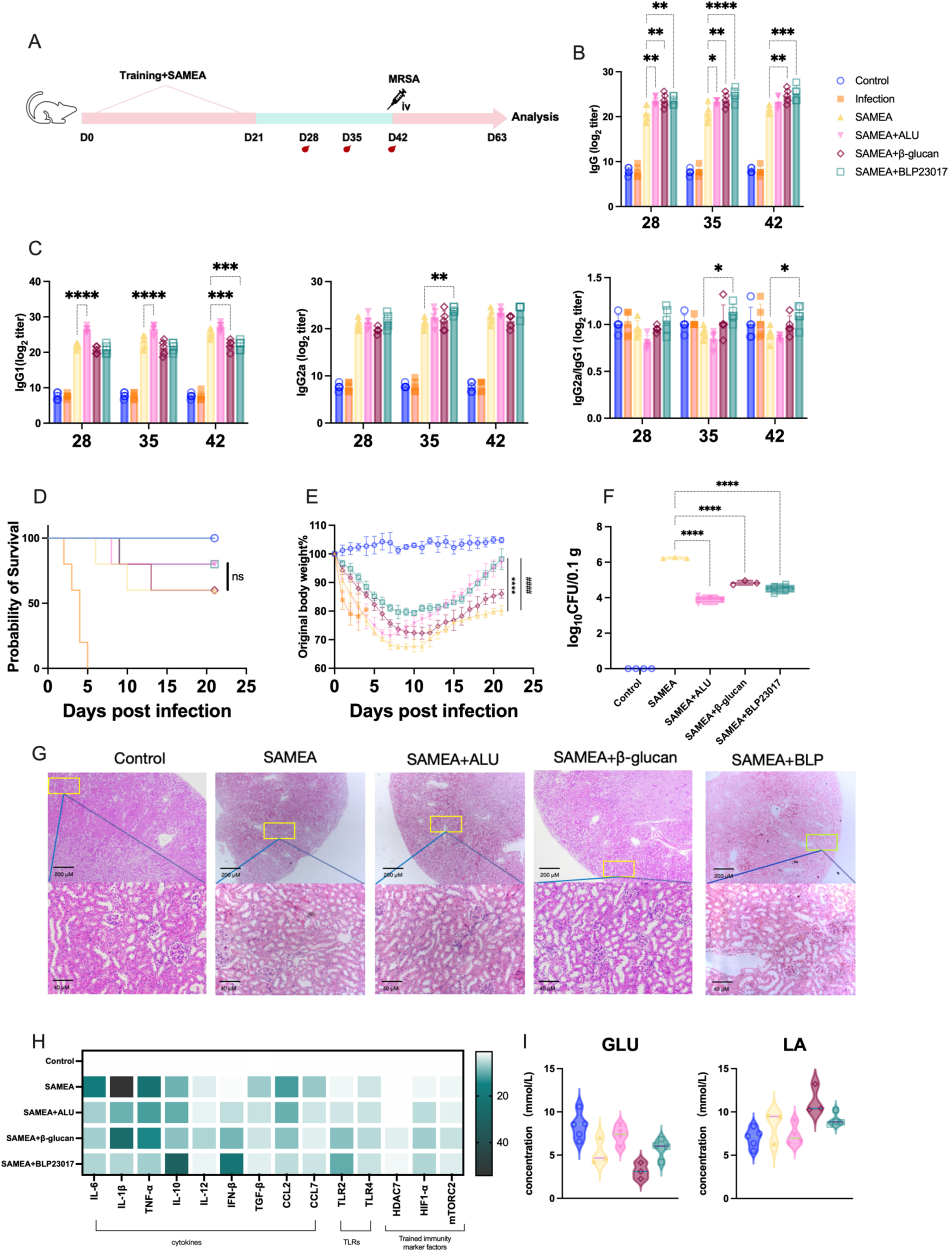
Bacterium-like particles (BLP) combined with the multi-epitope subunit vaccine-SAMEA provide better protection for mice. **(A)** Experimental design of BLP combined with two-dose subunit vaccine immunization against MRSA. **(B)** Specific IgG antibody levels in serum on days 28, 35, and 42 after immunization. **(C)** IgG1 antibody levels, IgG2a antibody levels, and the ratio of IgG2a/IgG in serum on days 28, 35, and 42. **(D)** The survival of mice after MRSA challenge. **(E)** The body weight change in mice after MRSA challenge. **(F)** The bacterial load of MRSA in the kidney on day 63. **(G)** Histological HE image of mice in the kidney on day 63. Scale bars, 200 and 40 µm. **(H)** The mRNA relative expression levels of IL-6, IL-1β, TNF-α, IL-10, IL-12, IFN-β, TGF-β, CCL2, CCL7, TLR2, TLR4, HDAC7, mTORC2, and HIF1-α on day 63 in kidney was measured by RT-qPCR. **(I)** The levels of glucose (GLU) and lactic acid (LA) in serum on day 63 (****/#### p<0.0001, *** p<0.001, ** p<0.01, * p<0.05). NS indicates that there is no significant difference between the two groups.. The results of qPCR were expressed using the 2^−ΔΔCT^ value.

To enhance the subunit vaccine SAMEA and improve its protective role against MRSA, we co-immunized with BLP in combination with SAMEA. Mice were immunized with a two-dose SAMEA combined with BLP on days 0 and 21; MRSA challenge via intravenous injection was performed on day 42 after the first immunization (**Figure 1A**), and we monitored antibody levels at regular intervals. The SAMEA-specific IgG antibody titers after two immunizations with or without the combination of ALU, β-glucan, and BLP are displayed as endpoint dilution titer in **Figure 1B**. The results showed that BLP significantly elevated SAMEA-specific IgG antibody levels at all three monitored time points (*p* < 0.01) (**Figure 1B**). When IgG2a/IgG1 > 1, the response is biased toward a Th1 cell immune response; the opposite is true for Th2 (59). Further subtype analysis showed that compared to the SAMEA group, the levels of IgG1 in the SAMEA+ALU group were increased, and the ratio of IgG2a/IgG1 was decreased. The ratio of IgG2a/IgG1 in the SAMEA+BLP group is increased (**Figure 1C**). The above data show that BLP induced higher levels of IgG antibodies and a more balanced Th response.

Subsequently, we evaluated whether BLP enhanced the vaccine’s protective effect against MRSA. We monitored the status of the mice 21 days after infection. The survival rates of mice in the SAMEA+BLP group and the SAMEA+ALU group were increased from 60% to 80% compared to the SAMEA group (**Figure 1D**). Changes in mouse body weight showed that mice in the BLP group had the slowest weight loss and recovered more quickly from day 0 after the MRSA infection (**Figure 1E**). Bacterial loads in mouse kidneys revealed that SAMEA+ALU, SAMEA+β-glucan, and SAMEA+BLP significantly reduced bacterial loads (*p* < 0.0001) compared to the SAMEA group (**Figure 1F**). Kidney histopathological sections also showed that, relative to the SAMEA alone group, BLP significantly reduced pathological damage and renal tubular vacuolization, which was attributed to the increased renal microsomes in the kidney caused by MRSA infection (**Figure 1G**).

Furthermore, we analyzed major cytokine and TLR transcript levels in mouse kidneys, finding that in combination with BLP, mouse kidney tissues exhibited superior inflammation resolution compared to the SAMEA group. Specifically, compared to the SAMEA group, we observed significantly lower transcript levels of IL-6, IL-1β, TNF-α, TGF-β, CCL2, and CCL7 and the highest transcript levels of IL-10 and IFN-β in the SAMEA+BLP group. TLR2 expression was significantly upregulated in all three groups compared to the SAMEA group, with the highest levels in the SAMEA+BLP group. Similarly, the SAMEA+BLP group showed the highest HIF-1α transcript levels in mouse kidneys (**Figure 1H**, **Supplementary Figures S1**, **S2**). To initially assess whether BLP induced changes in glycolysis levels, we examined serum levels of GLU and LA. The results show that the SAMEA+BLP group serum GLU and LA levels changed, but not to a significant degree (**Figure 1I**). All in all, BLP significantly improves vaccine immunization efficacy, elicits a more rapid organismal response to MRSA infection.

### 3.2 BLP-induced trained immunity also enhanced the protective effect of one dose of SAMEA vaccination

**Figure 2:**
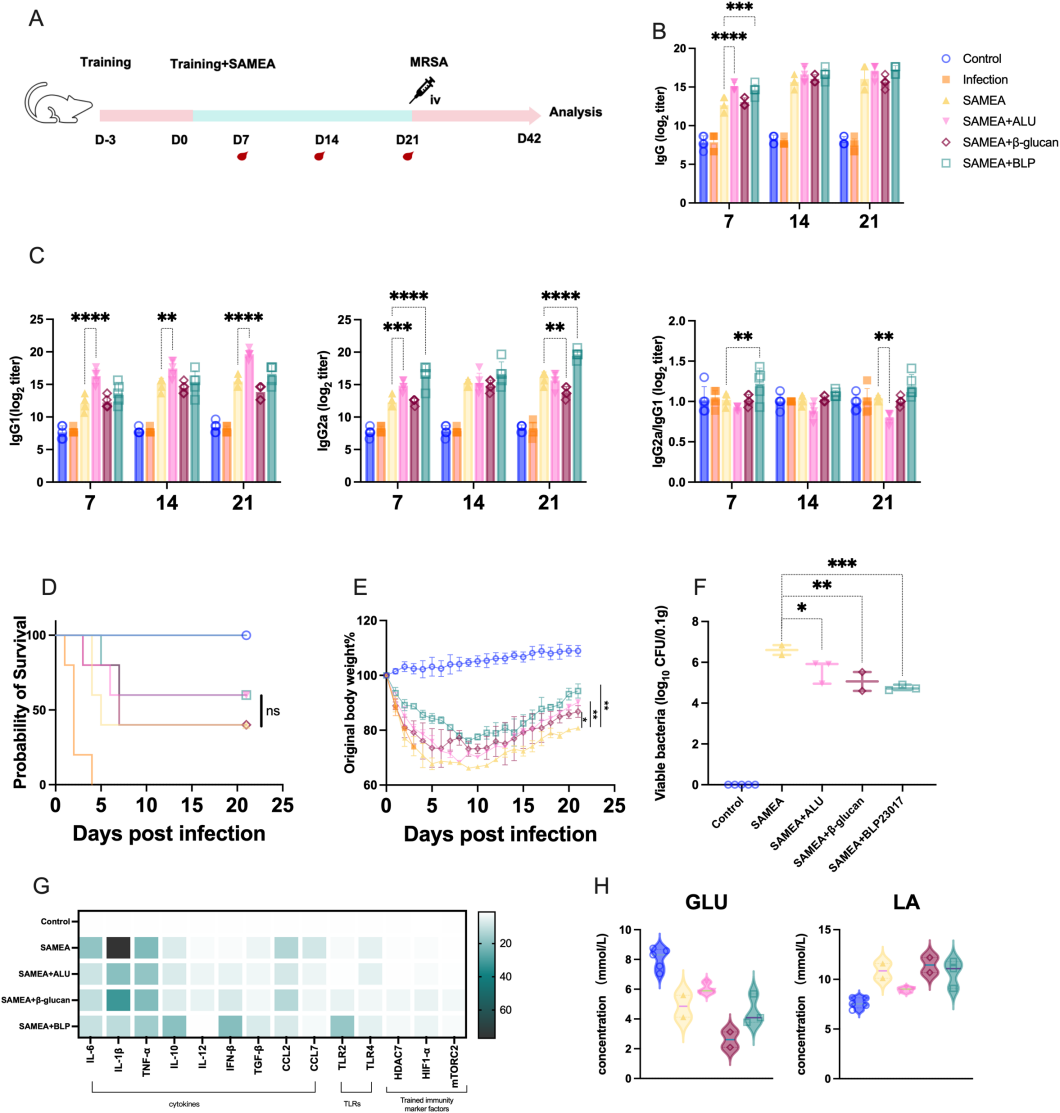
BLP combined with one dose of SAMEA also provides better protection for mice against MRSA infection. **(A)** Experimental design of BLP combined with one-dose subunit vaccine immunization against MRSA. **(B)** Specific IgG antibody levels in serum on days 7, 14, and 21. **(C)** IgG1 and IgG2a antibody levels and the ratio of IgG2a/IgG1 in serum on days 7, 14, and 21 after immunization. **(D)** The survival of mice after MRSA challenge. **(E)** The body weight change in mice after MRSA challenge. **(F)** The bacterial load of MRSA in the kidney on day 42. **(G)** Relative expression levels of cytokines and trained immunity-related indices in the mouse kidney on day 42 of IL-6, IL-1β, TNF-α, IL-10, IL-12, IFN-β, TGF-β, CCL2, CCL7, TLR2, TLR4, HDAC7, mTORC2, and HIF1-α were measured by RT-qPCR. **(H)** The levels of GLU and LA in serum on day 42 (**** p<0.0001, *** p<0.001, ** p<0.01, * p<0.05). NS indicates that there is no significant difference between the two groups. The results of qPCR were expressed using the 2^−ΔΔCT^ value.

We next established a one-dose immunization protocol with SAMEA to evaluate the adjuvant potential of BLP (**Figure 2A**). We also assessed whether a single dose of BLP could enhance antibody production. Dynamic monitoring revealed that the SAMEA+BLP group showed elevated IgG and IgG2a levels compared to the SAMEA group, with a significant difference observed only on day 7 (*p* < 0.001) (**Figure 2B**). Furthermore, the IgG2a/IgG1 ratio indicated a Th1-biased response in the SAMEA+BLP group, while the SAMEA+ALU group showed a Th2-biased response (**Figure 2C**).

Following intravenous MRSA challenge on day 21, mice were monitored for 21 days. The SAMEA+BLP group showed an improved survival rate of 60%, compared to 40% in the SAMEA group, and a similar rate to the SAMEA+ALU group (**Figure 2D**). Mice in the SAMEA+ALU (*p* < 0.01), SAMEA+β-glucan (*p* < 0.05), and SAMEA+BLP (*p* < 0.01) groups exhibited reduced body weight loss and better final weight retention compared to the SAMEA group. Notably, the SAMEA+BLP group showed less weight loss than the SAMEA+ALU group and recovered more rapidly (**Figure 2E**). Bacterial load analysis revealed that the SAMEA+BLP group had significantly lower kidney bacterial burdens than the SAMEA, SAMEA+ALU, and SAMEA+β-glucan groups (**Figure 2F**).

We further analyzed immune-related cytokines and TLR expression in mouse kidneys by qPCR at day 21 post-MRSA challenge. Compared to the SAMEA group, the SAMEA+BLP group showed significantly lower transcript levels of IL-6, IL-1β, CCL2, and CCL7, and significantly higher levels of IL-10 and IFN-β. TLR2 expression was significantly upregulated in the SAMEA+BLP group, while TLR4 expression remained unchanged (**Figure 2G**, **Supplementary Figures S3**, **S4**). In addition, serum GLU and LA levels showed no notable changes in this group (**Figure 2H**).

### 3.3 BLP induces trained immunity in RAW264.7 macrophages

**Figure 3:**
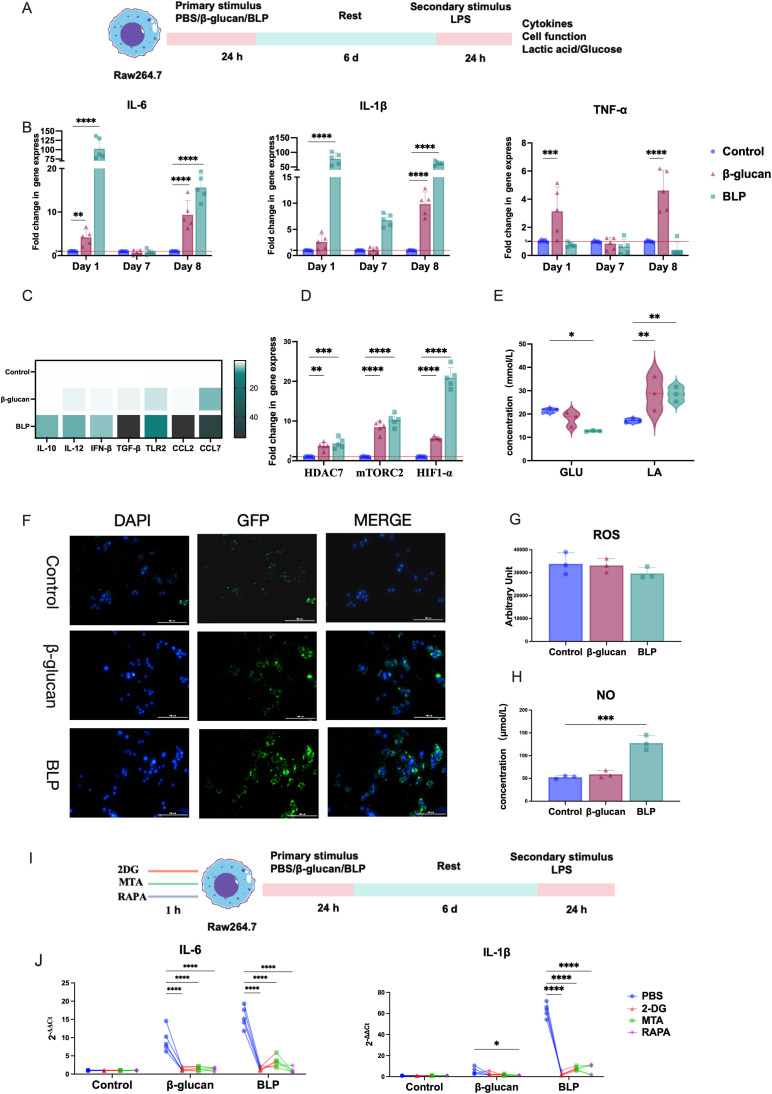
BLP induced trained immunity in Raw264.7 cells. **(A)** Experimental design of the trained immunity model *in vitro*. **(B)** The mRNA relative express levels of IL-6, IL-1β, and TNF-α on D1, D7, and D8 (*n* = 5). **(C)** Heatmap of the mRNA relative expression levels of IL-10, IL-12, IFN-β, TGF-β, TLR2, CCL2, and CCL7 on D8 (*n* = 5). **(D)** The mRNA relative expression levels of HDAC7, mTORC2, and HIF1-α on D8 (*n* = 5). The levels of **(E)** GLU and LA; **(G)** ROS; **(H)** NO in the cell supernatant on D8 (*n* = 3). **(F)** Raw264.7 cells on D7 were obtained and rested overnight before being co-cultured with *E. coli* pET28a-GFP/Rosetta for 1 h. A fluorescence microscope determines phagocytosis of Raw264.7 cells from each group. Scale bars, 100 µm. **(I)**. Inhibition experimental design of the trained immunity model *in vitro*. **(J)** After the action of an inhibitor, the mRNA relative expression levels of IL-6, IL-1β, and TNF-α on D8 were measured by RT-qPCR (****p < 0.0001, ***p < 0.001, **p < 0.01, *p < 0.05). The results of qPCR were expressed using the 2^−ΔΔCT^ value.

To assess whether BLP induces trained immunity in RAW264.7 cells, we established a trained immunity model following established protocols. Cells were first exposed to β-glucan or BLP, then rested and subsequently stimulated with LPS to evaluate macrophage responses (**Figure 3A**). Trained immunity is characterized by a memory-like phenotype and enhanced responses upon secondary stimulation. Thus, key cytokines associated with trained immunity (IL-6, IL-1β, and TNF-α) were measured. Following BLP treatment (day 1), IL-6 and IL-1β levels were significantly elevated, returned to baseline by day 7, and were again significantly upregulated upon LPS stimulation on day 8 (IL-6: *p* < 0.05; IL-1β: *p* < 0.0001) (**Figure 3B**). In contrast, TNF-α showed no significant change after primary stimulation and was reduced following secondary stimulation. To better characterize the inflammatory profile, we expanded the cytokine panel (**Figure 3C**, **Supplementary Figure S5**) and observed significantly increased transcript levels of IL-10, IL-12, IFN-β, TGF-β, TLR2, CCL2, and CCL7 in the BLP group compared to controls on day 8. These results suggest that BLP-induced trained immunity is associated with enhanced and diversified cytokine responses upon secondary challenge.

Previous studies have shown that epigenetic and metabolic reprogramming of macrophages through the Akt-mTOR-HIF-1α pathway is crucial for trained immunity (60). To explore the phenotypes of BLP-induced trained immunity, we assessed the transcript levels of HDAC7, mTORC2, and HIF-1α, along with GLU consumption and LA production. After secondary stimulation, the BLP group exhibited significantly higher expression of HDAC7, mTORC2, and HIF-1α (*p* < 0.01) compared to controls (**Figure 3D**). Additionally, BLP significantly increased GLU consumption (*p* < 0.05) and LA production (*p* < 0.01) (**Figure 3E**). These findings suggest that BLP-induced trained immunity may involve metabolic reprogramming.

Macrophage function is highly correlated with its ability to fight infection, and the proinflammatory profile of activated macrophages is usually associated with antimicrobial purposes (61). We investigated the activation status of macrophages after trained immunity by measuring their phagocytic activity, ROS, and NO levels. Immunofluorescence images showed more *E. coli* in BLP-induced macrophages (**Figure 3F**) than in the control group, showing that the phagocytic capacity was significantly increased. Meanwhile, the BLP-induced trained immunity exhibited decreased ROS levels and increased NO levels (*p* < 0.001) (**Figures 3G, H**). The above data suggest that BLP-induced trained immunity may be mediated through modulation of cell function.

To confirm the roles of metabolic and epigenetic reprogramming in BLP-induced trained immunity, macrophages were pretreated with the glycolysis inhibitor 2-DG, the histone methylation inhibitor MTA, and the mTOR inhibitor rapamycin (RAPA) (**Figure 3I**). BLP stimulation elevated IL-6 and IL-1β levels in RAW264.7 cells, but these responses were attenuated to varying degrees by all three inhibitors (**Figure 3J**). These results indicate that BLP-induced trained immunity is mediated through glycolysis, epigenetic regulation, and mTOR-dependent pathways.

### 3.4 BLP-induced trained immunity protected mice against *S. aureus* infection

**Figure 4:**
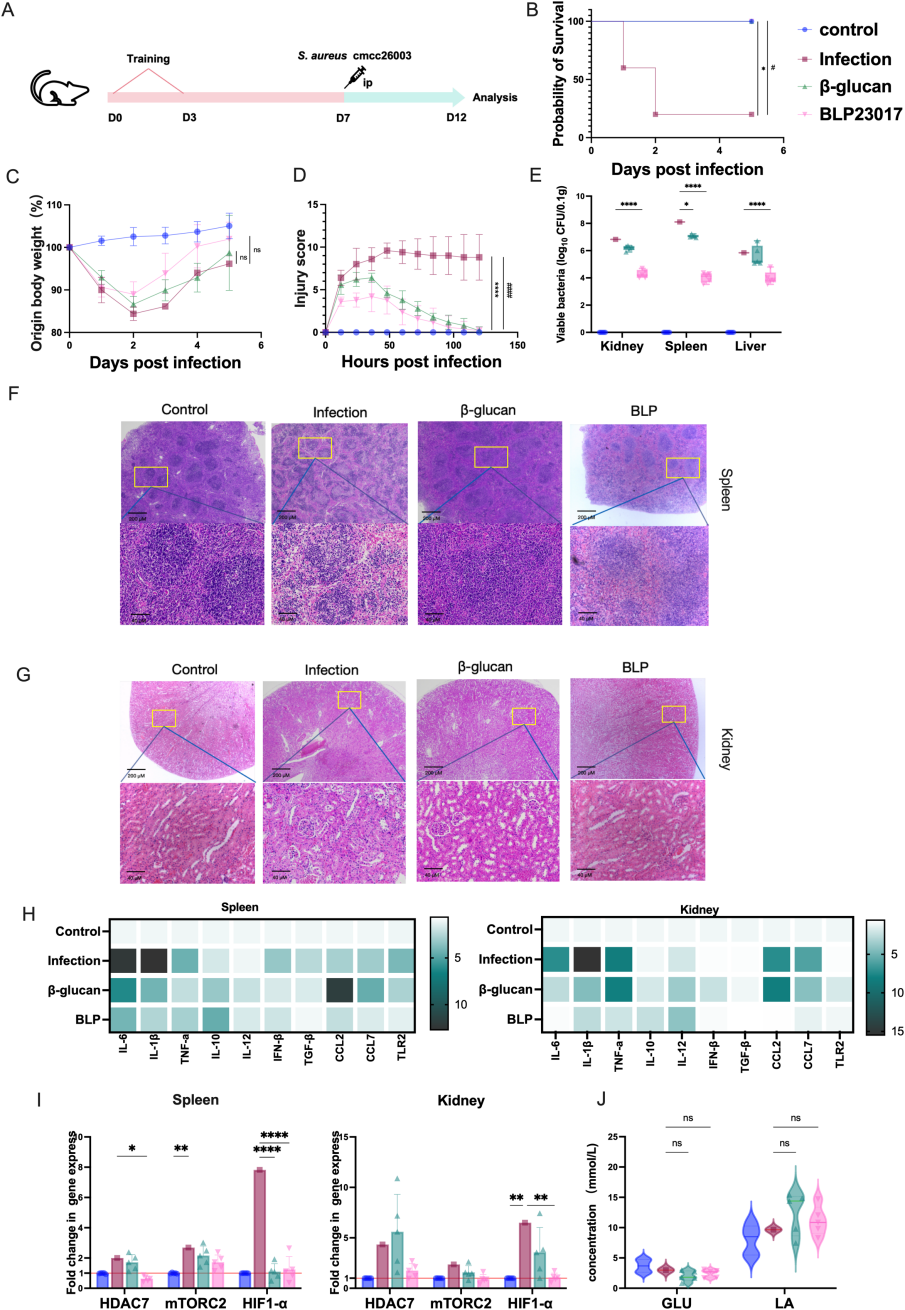
Trained immunity induced by BLP rapidly protected mice infected with *S. aureus*. **(A)** Experimental design of trained immunity model *in vivo*. **(B)** The survival of mice after *S. aureus* challenge. **(C)** The body weight change in mice after *S. aureus* challenge. **(D)** The injury score of mice after *S. aureus* challenge. **(E)** The bacterial load of *S. aureus* in the spleen, kidney, and liver on D12. **(F)** Histological HE image of the mouse spleen on D12. Scale bars, 100 µm. **(G)** Histological HE image of the mouse kidney on D12. Scale bars, 100 µm. **(H)** Heatmap of the mRNA relative expression levels of IL-10, IL-12, IFN-β, TGF-β, TLR2, CCL2, and CCL7 on D12 in the spleen and kidney (*n* = 5). **(I)** The mRNA relative expression level of HDAC7, mTORC2, and HIF1-α on D12 in the spleen and kidney was measured by RT-qPCR. **(J)** The levels of GLU and LA in serum on D12 (****/#### p<0.0001, *** p<0.001, ** p<0.01, */# p<0.05). NS indicates that there is no significant difference between the two groups.. The results of qPCR were expressed using the 2^−ΔΔCT^ value.

Because BLP can play a role in inducing RAW264.7 cells to produce trained immunity, we hypothesized that the trained immunity induced by BLP may also play a role in mice. Accordingly, mice trained with or without BLP and β-glucan were intraperitoneally injected with *S. aureus* CMCC26003 to determine their survival (**Figure 4A**). The results showed that trained immunity induced by BLP increased the survival rate of wild-type mice from 20% to 100% (**Figure 4B**). BLP is highly protective against weight loss and increased injury in mice. Mice in the BLP group lost less body weight and had a faster rate of recovery relative to the infected group (*p* > 0.05) (**Figure 4C**). The injury score of BLP-treated mice was less than the infected mice and β-glucan treatment mice (*p* < 0.0001) (**Figure 4D**). The above data suggest that trained immunity induced by BLP in mice increases survival and decreases body weight changes and body damage.

The mice trained with BLP also showed lower bacterial burdens in the kidneys, spleens, and livers (*p* < 0.0001) compared to the infection group (**Figure 4E**). Spleen and kidney injuries were reduced in the BLP group of mice compared with the infected group. The red and white marrow boundaries were clear, and no pathological changes such as megakaryocytic hyperplasia were seen (**Figure 4F**). Cytokine transcript analyses in the spleens and kidneys revealed a diminished inflammatory response in mice pretreated with BLP. Specifically, spleen cytokine levels, including IL-6, IL-1β, TNF-α, IFN-β, TGF-β, CCL7, and TLR2, were reduced in BLP groups relative to the infected group (**Figure 4G**). Kidney cytokine levels in the BLP group were lower than those in the infected group, whereas levels of CCL2, CCL7, and TNF-α in the β-glucan group tended to be higher than those in the infected group (**Figure 4H**, **Supplementary Figures S6**, **S7**). The above data suggest that trained immunity induced by BLP in mice can reduce tissue bacterial load, pathological injury, and inflammatory response in mice.

Additionally, in spleen tissue, compared to the control group, the levels of HDAC7 (*p* < 0.05) and HIF-1α (*p* < 0.001) were notably lower in BLP-treated mice, but the levels of mTORC2 showed no significant changes (*p* > 0.05) (**Figure 4I**). In the kidneys, the levels of HIF-1α were significantly reduced in the BLP group compared to the infected group (*p* < 0.01). Serum GLU shows a downward trend while LA shows an upward trend, but the difference is not significant (*p* > 0.05) (**Figure 4J**). Overall, pretreatment of mice with BLP provided effective protection against *S. aureus* infection.

### 3.5 BLP-induced mouse peritoneal macrophages mount a rapid immune response upon secondary challenge

**Figure 5:**
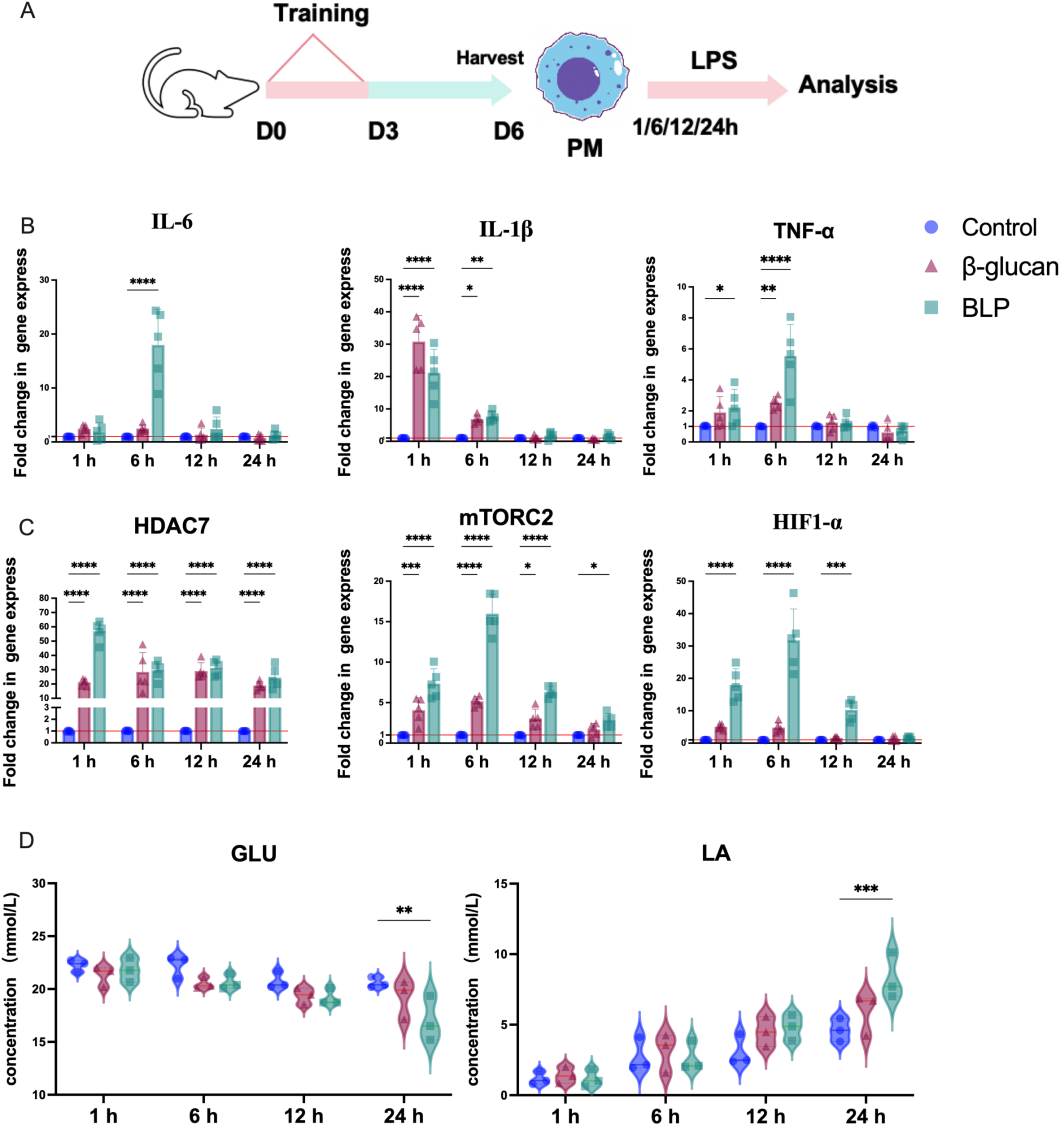
**(A)** Schematic showing trained immunity in the stimulated with LPS in mouse peritoneal macrophages. The mRNA relative expression levels of **(B)** IL-6, IL-1β, and TNF-α; **(C)** HDAC7, mTORC2, and HIF1-α in 1, 6, 12, and 24 h were measured by RT-qPCR (*n* = 5). The levels of **(D)** GLU and LA in the cell supernatant at 1, 6, 12, and 24 h (****p < 0.0001, ***p < 0.001, **p < 0.01, *p < 0.05). The results of qPCR were expressed using the 2^−ΔΔCT^ value.

Both *in vitro* and *in vivo* experiments have shown that BLP immunization could induce increased proinflammatory cytokines and chemokines after the secondary challenge against heterologous pathogens. We hypothesized that the trained immunity induced by BLP may also mount a faster and greater response in mouse peritoneal macrophages. For this purpose, the peritoneal macrophages of mice trained with or without BLP and β-glucan were extracted and re-stimulated for 24 h *in vitro* with LPS (**Figure 5A**). We then monitored the transcripts of typical trained immunity markers under continuous LPS stimulation for 1, 6, 12, and 24 h. Here, BLP immunization induced elevated proinflammatory cytokines of IL-6, IL-1β, and TNF-α release rapidly compared to that of the control in peritoneal macrophages of mice in 6 h after (**Figure 5B**). The significantly elevated expression of HDAC7, mTORC2, and HIF-1α persisted in the BLP group in 1, 6, and 12 h (*p* < 0.05) (**Figure 5C**). Analysis of GLU and LA concentrations in the cell supernatant showed that the decreased levels of GLU and elevated levels of LA were consistently elevated in the β-glucan and BLP groups. Moreover, the decreased levels of GLU and the elevated levels of LA were significantly higher inside BLP-treated cells than the control group in 24 h (**Figure 5D**). In summary, after induction with BLP, LPS stimulation elicits a rapid response in macrophages, characterized by increased secretion of proinflammatory cytokines, peaking at 6 h. Meanwhile, BLP-induced trained immunity exhibits enhanced levels of epigenetic and metabolic-related factors and glycolysis.

### 3.6 BLP induced trained immunity by upregulating the IL-6-JAK-STAT3 signaling pathway

**Figure 6:**
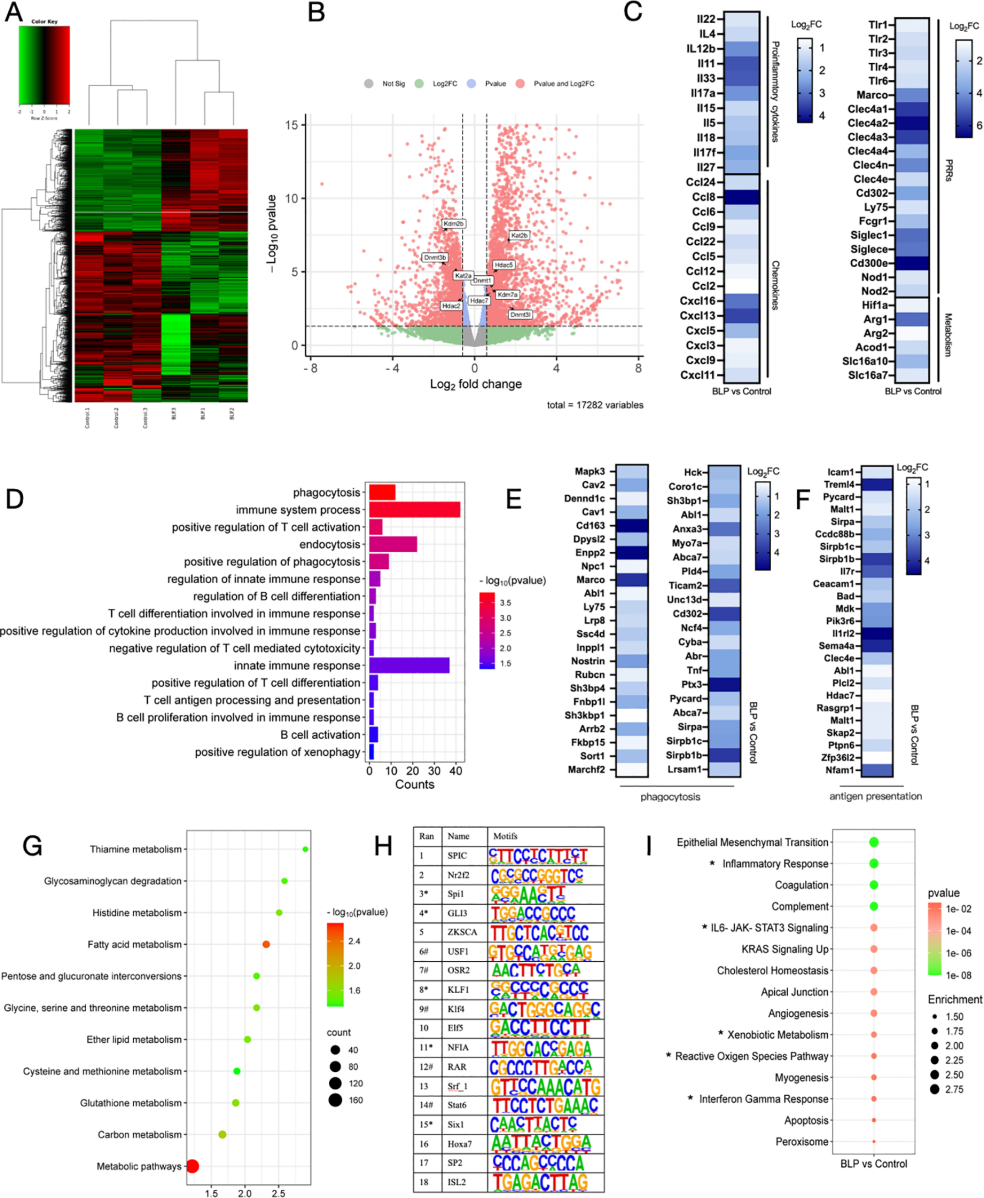
Mouse peritoneal macrophages stimulated with LPS for 6 h were analyzed for RNA-seq. **(A)** Heatmap plot of differentially expressed genes between the Control group and the BLP group. **(B)** Volcano plot of differentially expressed genes between the Control group and the BLP group. Differential expression of epigenetically related genes is labeled. **(C)** Heatmap of related upregulated genes of proinflammatory cytokines, chemokines, PRRs, and metabolism. **(D)** Immunity and phagocytosis-related GO function analysis histogram of differentially expressed genes. Heatmap of related upregulated genes of **(E)** phagocytosis and **(F)** antigen presentation-related genes. **(G)** Metabolism-related pathway KEGG enrichment. **(H)** Comparative transcription factor enrichment analysis of differentially expressed genes using the HOMER package (* indicates transcription factors involved in the epigenetic reprogramming, # indicates transcription factors involved in the innate immune response). **(I)** Gene set enrichment analysis (GSEA) using the Molecular Signatures Database (MSigDB) Hallmark gene set collection. Differentially expressed gene sets in BLP-treated monocytes versus controls.

Based on the findings that BLP induced trained immunity and provided heterogeneous protection, we collected macrophages from each group that had been continuously stimulated with LPS for 6 h and conducted RNA-seq analyses to elucidate the underlying mechanisms. Heatmap analysis revealed distinct gene expression profiles for each group (**Figure 6A**). Using DESeq2, we identified 1,960 DEGs in the BLP group relative to the control group, comprising 927 upregulated and 1,023 downregulated genes (**Figure 6B**). At the same time, we found changes in epigenetically related upregulated genes such as DNA (cytosine-5)-methyltransferase genes (Dnmt1, Dnmt3c, and Dnmt3l), histone deacetylase (Hdac7 and Hdac5), lysine (K)-specific demethylase 7A (kdm7a), and K (lysine) acetyltransferase 2B (kat2b) (**Figure 6B**). The above data suggest that BLP-induced trained immunity may have influenced epigenetic changes, but the exact role remains unclear. After BLP training, the inflammatory cytokines (IL22, Il4, etc.), chemokines (Ccl2, Cxcl3, Cxcl5, etc.), PRRs (TLR1-4, Nod1, Nod2, etc.), and metabolism-related factors (Hif-1α, Arg1, Acod1, etc.) were elevated after secondary stimulation (**Figure 6C**).

GO analyses indicated that BLP upregulated biological processes associated with immunity, such as immune system process, positive regulation of cytokine production involved in immune response, and innate immune response. Also, processes related to phagocytosis, such as phagocytosis, positive regulation of phagocytosis, positive regulation of xenophagy, and endocytosis, are upregulated (**Figure 6D**). Related genetic changes such as MAPK3, CAV, and CD163 are shown in **Figure 6E**. BLP-induced trained immunity significantly elevated macrophage phagocytosis and altered macrophage function, similar to the results we obtained in RAW264.7 cells. There was also a significant enrichment of upregulated genes associated with antigen presentation, such as Icam1, Malt1, and Mdk (**Figure 6F**). The results showed that BLP-induced trained immunity significantly activated the innate immune response and modulated cytokines to fight against exogenous infections. This also suggests that our BLP-induced trained immunity may enhance the immunization effect of the vaccine.

KEGG analyses indicated that a large number of genes are enriched in metabolic pathways, including the process of glycolysis, as well as amino acid metabolism and fatty acid metabolism. The enrichment of genes in the glycolytic pathway also corroborates our previous findings (**Figure 6G**). Next, we carried out a comparative transcription factor (TF) enrichment analysis of upregulated DEGs in the BLP and control groups using the HOMER package. Motif analysis was conducted to explore which TFs are involved in regulating the epigenetic modifications associated with BLP training. As shown in **Figure 6H**, the Sfpi1 motif was found as a regulator of 40.62% of upregulated genes for the first rank genes. It is a function including histone deacetylase binding activity and regulation of myeloid leukocyte-mediated immunity. On the other hand, motifs associated with KLF1 contributed to 35.99% of upregulated genes; they act upstream of or within chromatin remodeling. GSEA using the Hallmark gene set collection from the Molecular Signatures Database (MSigDB) indicated that BLP training leads to the upregulation of various genes related to Inflammatory Response, IL-6-JAK-STAT3 Signaling, Cholesterol Homeostasis, Xenobiotic Metabolism, and Interferon-gamma Response (**Figure 6I**, **Supplementary Figure S8**). Based on the above data, we hypothesized that BLP may induce trained immunity by activating the JAK-STAT3 pathway through the activation of the TLR2 receptor secretion of IL-6, which regulates HIF-1α and subsequently modulates macrophage metabolic reprogramming and cellular function.

## 4 Discussion

The rise of MDR bacteria poses a major challenge to modern healthcare (62). Coinfection with MRSA significantly increases mortality risk in both adults and children (3). A potential solution lies in the development of TIbV vaccines, which leverage trained immunity to prevent coinfections and combat antimicrobial resistance driven by the proliferation of MDR bacteria. In this study, we hypothesized that the induction of trained immunity might explain how BLP enhances immune responses, positioning BLP as a novel TIbV vaccine vector candidate. Therefore, our study aimed to elucidate the mechanisms by which BLP boosts vaccine immunity, with a particular focus on its potential to induce trained immunity. Our findings suggest that combining BLP with the vaccine provides more robust protection against MRSA infection in mice, offering early protection and facilitating faster recovery in later stages. BLP induced a trained immunity phenotype in RAW264.7 cells and isolated peritoneal macrophages, demonstrating heterologous protection against *S. aureus* infection in mice. Supported by transcriptome analysis, we hypothesized that BLP induces trained immunity through activation of the JAK-STAT3 pathway via TLR2 receptor stimulation, leading to IL-6 secretion. This pathway regulates HIF-1α, subsequently modulating macrophage metabolic reprogramming and cellular function. Our results indicate that induction of trained immunity is a key mechanism through which BLP enhances immune responses. This finding underscores the potential of BLP as a vaccine vector for developing TIbV vaccines aimed at preventing coinfections and combating antimicrobial resistance associated with MDR bacteria.

In this study, we found that when BLP was used as an immunopotentiator in combination with SAMEA, it significantly enhanced the protective efficacy of SAMEA against intravenous challenge with *S. aureus* USA26003. BLP appeared to induce a rapid immune response, suggesting its potential to activate innate immunity. Based on this, we hypothesized that BLP may act as an effective inducer of trained immunity and designed our study accordingly. Moreover, immunization with SAMEA alone elicited a strong antibody response, likely due to its design as a multi-epitope antigen capable of effectively activating T- and B-cell responses. Previous studies have also shown that multi-epitope vaccines can elicit robust antibody responses even in the absence of adjuvants (63, 64). Previous studies on trained immunity have primarily focused on using pathogen-derived PAMPs such as LPS or β-glucan (19, 65). In our study, we investigated the relationship between peptidoglycan backbone BLP from the probiotic *Levilactobacillus* and trained immunity. Trained immunity refers to the enhanced responsiveness of innate immune cells to subsequent challenges following initial exposure (19). Our findings demonstrate that BLP can induce trained immunity. Our analysis of cytokine production in RAW264.7 macrophages stimulated by BLP, followed by re-stimulation with LPS, reveals a heightened reactive state and features indicative of immune memory—a trained immunity signature phenotype. Additionally, BLP-treated RAW264.7 macrophages exhibit altered epigenetic and metabolic reprogramming, akin to the effects observed with the trained immunity activator β-glucan. Upon LPS re-stimulation, these macrophages notably increase expression of key proinflammatory cytokines such as IL-6, IL-1β, IL-10, IL-12, IFN-β, and TGF-β, as well as markers like TLR2, CCL2, and CCL7, consistent with established research (43, 66). Moreover, pretreatment of mice with protein-free BLP confers effective protection against *S. aureus* infection, demonstrating a heterologous protection profile *in vivo*. This evidence supports the effectiveness of BLP-induced trained immunity in mice, highlighting its potential advantage in vaccine development. Through *in vitro* and *in vivo* experiments, we established that BLP treatment induces trained immunity. Macrophages and monocytes, crucial in host defense against infections (61), show enhanced immune memory following gavage administration of our stimulant. Notably, secondary stimulation leads to significantly elevated expression levels of HADC7, mTOR, and HIF-1α in peritoneal macrophages, indicating the induction of trained immunity. Studies on subcutaneous BCG vaccination have shown similar induction of memory alveolar macrophages (AMs) and trained immunity in the lung (67). Likewise, oral administration of β-glucan enhances the activities of peritoneal macrophages, including phagocytosis, candidacidal function, and IL-1 production in mice (68). These findings underscore that trained mucosal-resident macrophages act as frontline defenders upon pathogen entry, bolstered by recruited trained circulating monocytes (69). In summary, whether in peritoneal macrophages from mice or cultured RAW264.7 cells, BLP consistently induces immune memory production and protects against heterologous infections in mice. Thus, BLP-induced rapid recall responses to infection facilitate bacteria elimination, preventing organ damage and enhancing overall mouse survival, underscoring its role in trained immunity induction.

Innate immune memory has been demonstrated to involve significant reprogramming of chromatin marks (18), particularly histone 3 lysine 4 trimethylation, which is pivotal in defining active promoters of proinflammatory cytokine genes associated with a trained immunity phenotype. Previous studies have shown that β-glucan-induced trained immunity is epigenetically regulated at the histone level (21). Our findings reveal that upon secondary stimulation with BLP, HDAC7 expression was markedly upregulated in both RAW264.7 macrophages and peritoneal macrophages. Additionally, inhibition of histone demethylases significantly reduced the expression of trained immunity markers induced by β-glucan and BLP in RAW264.7 cells. A similar effect was observed in a study on β-glucan-induced trained immunity in dogs, where inhibition of methyltransferases by MTA led to a significant reduction in IL-6 secretion (43). These results underscore that BLP induces trained immunity through an epigenetic mechanism akin to β-glucan. Transcriptomic analysis further demonstrated that BLP-induced trained immunity involves upregulation of HDAC7 and several epigenetically related genes. The alteration in chromatin marks likely forms the basis of BLP-induced trained immunity, although the extent of its role remains to be fully elucidated.

Besides inflammatory cytokines, phagocytosis by macrophages represents another defense mechanism against infection in trained immunity (44, 70). Consistently, macrophages induced by BLP showed increased phagocytic capacity compared to the control group, evidenced by higher uptake of *E. coli*. Moreover, BLP-induced trained immunity was associated with decreased ROS levels and increased NO production. Pan et al. demonstrated that SPIO, known for inducing trained immunity, enhances macrophage phagocytic activity (44). Our findings align with previous research where cells primed with *Lactobacillus plantarum* exhibited reduced mitochondrial respiration and ROS production upon secondary stimulation (71). These results affirm that BLP induces trained immunity by modulating macrophage function. GO analysis revealed that numerous upregulated genes were enriched in pathways related to macrophage phagocytosis, suggesting that BLP may confer heterologous protective effects primarily through trained immunity-mediated enhancement of macrophage function. Cearra et al. also demonstrated increased expression of phagocytosis-related genes upon secondary exposure to bacteria (71), underscoring this cellular functional change as a key mechanism of BLP-induced trained immunity.

One of the key mechanisms governing trained immunity is the metabolic reprogramming of innate immune cells. Trained monocytes usually show elevated GLU uptake and increased LA production, signaling a shift from oxidative phosphorylation to aerobic glycolysis, which is mediated through the Dectin-1/Akt/HIF-1α pathway (60). In our study, BLP-induced trained immunity significantly upregulated mTORC2 and HIF-1α expression, influencing GLU metabolism in Raw264.7 cells and mouse peritoneal macrophages. Moreover, inhibition of glycolysis using 2-DG and mTOR pathway inhibitors attenuated the immune memory response in macrophages. Some studies have suggested that inhibiting glycolysis with 2-DG during BCG-induced training also eliminates the enhanced cytokine production (72). Our transcriptome results also indicated that BLP-induced trained immunity exhibits changes in multiple metabolism-related pathways. The results confirm that the BLP induces trained immunity through regulation and changes in metabolic reprogramming through mTOR expression.

In this study, we identified potential molecules, regulators, and pathways involved in BLP-induced trained immunity through RNA-seq and bioinformatics analysis. Our hypothesis suggests that BLP activates the mTOR/HIF-1α pathway via TLR2, leading to regulation of cytokine release, subsequent activation of the JAK-STAT3 pathway, and impacting metabolic reprogramming and macrophage activation. We observed significant upregulation of TLR2 expression upon BLP activation, as peptidoglycan, the main component of BLPs, acts as a TLR2 ligand (8). Studies have highlighted TLR2 as essential for BLP-induced immunoprotection (73, 74). Activation of TLR2 by BLP induced trained immunity in macrophages, significantly enhancing IL-6 and IFN-γ expression upon secondary stimulation (75). Furthermore, TLR2 activation influences the release of various cytokines, including IFN-γ. Activation of the JAK-STAT3 signaling pathway, known to be triggered by IFN-γ and IL-6 (76), in turn influenced HIF-1α expression. We observed upregulation of HIF-1α in both RAW264.7 macrophages and peritoneal macrophages, with indications that Stat3 expression may be necessary for HIF-1 activity (77). These pathways collectively regulate macrophage metabolism and enhance phagocytic activity. While we propose a mechanistic pathway for BLP-induced trained immunity, its precise role requires further exploration.

The development of immunological memory through vaccination is crucial for combating infections. BLPs, characterized by ease of preparation, high safety, stability, and efficient delivery, serve as effective carriers for antigen presentation through surface display with anchor proteins (9). Previous studies from our laboratory have utilized BLPs as vaccine delivery vehicles, demonstrating efficacy in enhancing vaccine-induced immunity (12). Our study with BLP+SAMEA suggests rapid protection, potentially due to sustained and activated immune responses triggered by vaccination. These findings resonate with studies by Wimmers et al., indicating transcriptional and epigenetic reprogramming of innate immune cells post-influenza vaccination (33). Similarly, Paris et al. utilized β-glucan as an adjuvant to enhance specific immune responses to rabies vaccines (78). Our transcriptomic analysis revealed alterations in genes related to antigen presentation, suggesting that BLP, as a vaccine delivery system, significantly enhances vaccine efficacy through trained immunity mechanisms.

A previous study indicated that BLP prepared by *L. lactis* could stimulate DC maturation *in vitro*. The maturation of specialized antigen-presenting cells, such as DCs, is a crucial phase in the initiation of a targeted immune response (79). Therefore, the application of BLP as a vaccine delivery vehicle to construct vaccines that can induce both trained immunity and specific immunity has important application prospects in the next study. BLP holds promise in vaccine design and development, although many aspects remain to be elucidated. Our study underscores BLP’s effective induction of trained immunity. Unlike traditional activators that lack vaccine carrier capabilities, BLP’s potential in vaccine design allows for rapid, broad-spectrum protection through trained immunity activation. Nonetheless, our study has limitations; while we focused on BLP-induced trained immunity in macrophages, a more comprehensive assessment across other innate immune cells, such as DCs and NK cells, would be beneficial. Additionally, while our study indicates that BLP-induced trained immunity involves epigenetic reprogramming of macrophages, further mechanistic insights are required.

## 5 Conclusion

In conclusion, the peptidoglycan backbone of lactic acid bacteria—BLP—can induce a trained immunity in vitro mouse macrophage model, an in vivo mouse model, and ex vivo peritoneal macrophages. BLP-induced trained immunity is manifested by an increase in trained immunity signature cytokines, increased phagocytic capacity of macrophages, and metabolic reprogramming. Furthermore, BLP can also protect *S. aureus* 26003 in the absence of antigens. At the same time, BLP can enhance the protection effect of a subunit vaccine via trained immunity against MRSA infection. Additionally, transcriptome analysis revealed BLP-induced trained immunity by activating the mTOR/HIF-1α pathway via TLR2, leading to the regulation of cytokine release and the subsequent activation of the JAK-STAT3 pathway, hence impacting metabolic reprogramming and macrophage activation. Thus, the application of BLP as a vaccine delivery vehicle to construct vaccines that can induce both trained immunity and specific immunity has important application prospects in the next study.

## Data Availability

This raw RNA-seq data can be found here: www.ncbi.nlm.nih.gov, accession number 613PRJNA1269762.

## References

[1] Roberts RR Hota B Ahmad I Scott RD Foster SD Abbasi F. Hospital and societal costs of antimicrobial-resistant infections in a Chicago teaching hospital: implications for antibiotic stewardship. Clin Infect Diseases: an Off Publ Infect Dis Soc America. (2009) 49:1175–84. doi: 10.1086/605630, PMID: 19739972

[2] Sievert DM Ricks P Edwards JR Schneider A Patel J Srinivasan A. Antimicrobial-resistant pathogens associated with healthcare-associated infections: summary of data reported to the National Healthcare Safety Network at the Centers for Disease Control and Prevention 2009-2010. Infection Control Hosp Epidemiol. (2013) 34:1–14. doi: 10.1086/668770, PMID: 23221186

[3] Randolph AG Xu R Novak T Newhams MM Bubeck Wardenburg J Weiss SL. Vancomycin monotherapy may be insufficient to treat methicillin-resistant staphylococcus aureus coinfection in children with influenza-related critical illness. Clin Infect Diseases: an Off Publ Infect Dis Soc America. (2019) 68:365–72. doi: 10.1093/cid/ciy495, PMID: 29893805 PMC6336914

[4] Campfield B Chen K Kolls JK. Vaccine approaches for multidrug resistant Gram negative infections. Curr Opin In Immunol. (2014) 28:84–9. doi: 10.1016/j.coi.2014.02.002, PMID: 24637162 PMC4037349

[5] Khalid K Poh CL. The Promising Potential of Reverse Vaccinology-Based Next-Generation Vaccine Development over Conventional Vaccines against Antibiotic-Resistant Bacteria. Vaccines. (2023) 11:1264. doi: 10.3390/vaccines11071264, PMID: 37515079 PMC10385262

[6] Vollmer W Blanot D de Pedro MA. Peptidoglycan structure and architecture. FEMS Microbiol Rev. (2008) 32:149–67. doi: 10.1111/j.1574-6976.2007.00094.x, PMID: 18194336

[7] Bosma T Kanninga R Neef J Audouy SAL van Roosmalen ML Steen A. Novel surface display system for proteins on non-genetically modified gram-positive bacteria. Appl Environ Microbiol. (2006) 72:880–9. doi: 10.1128/AEM.72.1.880-889.2006, PMID: 16391130 PMC1352190

[8] Zhou X Gao M De X Sun T Bai Z Luo J. Bacterium-like particles derived from probiotics: progress, challenges and prospects. Front In Immunol. (2023) 14:1263586. doi: 10.3389/fimmu.2023.1263586, PMID: 37868963 PMC10587609

[9] Niu L Gao M Ren H De X Jiang Z Zhou X. A novel bacterium-like particles platform displaying antigens by new anchoring proteins induces efficacious immune responses. Front In Microbiol. (2024) 15:1395837. doi: 10.3389/fmicb.2024.1395837, PMID: 38841059 PMC11150769

[10] Audouy SAL van Roosmalen ML Neef J Kanninga R Post E van Deemter M. Lactococcus lactis GEM particles displaying pneumococcal antigens induce local and systemic immune responses following intranasal immunization. Vaccine. (2006) 24:5434–41. doi: 10.1016/j.vaccine.2006.03.054, PMID: 16757068

[11] Ramirez K Ditamo Y Rodriguez L Picking WL van Roosmalen ML Leenhouts K. Neonatal mucosal immunization with a non-living, non-genetically modified Lactococcus lactis vaccine carrier induces systemic and local Th1-type immunity and protects against lethal bacterial infection. Mucosal Immunol. (2010) 3:159–71. doi: 10.1038/mi.2009.131, PMID: 19924118 PMC2863133

[12] Guo Z Ren H Chang Q Liu R Zhou X Xue K. Lactobacilli-derived adjuvants combined with immunoinformatics-driven multi-epitope antigens based approach protects against Clostridium perfringens in a mouse model. Int J Biol Macromolecules. (2024) 267:131475. doi: 10.1016/j.ijbiomac.2024.131475, PMID: 38608984

[13] Netea MG Quintin J van der Meer JWM. Trained immunity: a memory for innate host defense. Cell Host Microbe. (2011) 9:355–61. doi: 10.1016/j.chom.2011.04.006, PMID: 21575907

[14] Zhu B Wu Y Huang S Zhang R Son YM Li C. Uncoupling of macrophage inflammation from self-renewal modulates host recovery from respiratory viral infection. Immunity. (2021) 54:1200–18. doi: 10.1016/j.immuni.2021.04.001, PMID: 33951416 PMC8192557

[15] Netea MG Joosten LAB van der Meer JWM. Hypothesis: stimulation of trained immunity as adjunctive immunotherapy in cancer. J Leukoc Biol. (2017) 102:1323–32. doi: 10.1189/jlb.5RI0217-064RR, PMID: 29018149

[16] Dimitrov I Flower DR Doytchinova I. AllerTOP–a server for in silico prediction of allergens. BMC Bioinf. (2013) 14 Suppl 6:S4. doi: 10.1186/1471-2105-14-s6-s4, PMID: 23735058 PMC3633022

[17] Khader SA Divangahi M Hanekom W Hill PC Maeurer M Makar KW. Targeting innate immunity for tuberculosis vaccination. J Clin Invest. (2019) 129:3482–91. doi: 10.1172/JCI128877, PMID: 31478909 PMC6715374

[18] Netea MG van der Meer JW. Trained immunity: an ancient way of remembering. Cell Host Microbe. (2017) 21:297–300. doi: 10.1016/j.chom.2017.02.003, PMID: 28279335

[19] Netea MG Domínguez-Andrés J Barreiro LB Chavakis T Divangahi M Fuchs E. Defining trained immunity and its role in health and disease. Nat Rev Immunol. (2020) 20:375–88. doi: 10.1038/s41577-020-0285-6, PMID: 32132681 PMC7186935

[20] Rodrigues MV Zanuzzo FS Koch JFA de Oliveira CAF Sima P Vetvicka V. Development of fish immunity and the role of β-glucan in immune responses. Molecules (Basel Switzerland). (2020) 25:5378. doi: 10.3390/molecules25225378, PMID: 33213001 PMC7698520

[21] Quintin J Saeed S Martens JHA Giamarellos-Bourboulis EJ Ifrim DC Logie C. Candida albicans infection affords protection against reinfection via functional reprogramming of monocytes. Cell Host Microbe. (2012) 12:223–32. doi: 10.1016/j.chom.2012.06.006, PMID: 22901542 PMC3864037

[22] de Laval B Maurizio J Kandalla PK Brisou G Simonnet L Huber C. C/EBPβ-dependent epigenetic memory induces trained immunity in hematopoietic stem cells. Cell Stem Cell. (2020) 26:657–74. doi: 10.1016/j.stem.2020.01.017, PMID: 32169166

[23] Théroude C Reverte M Heinonen T Ciarlo E Schrijver IT Antonakos N. Trained immunity confers prolonged protection from listeriosis. Front In Immunol. (2021) 12:723393. doi: 10.3389/fimmu.2021.723393, PMID: 34603295 PMC8484647

[24] Sánchez-Ramón S Conejero L Netea MG Sancho D Palomares Ó. Subiza JL. Trained immunity-based vaccines: A new paradigm for the development of broad-spectrum anti-infectious formulations. Front In Immunol. (2018) 9:2936. doi: 10.3389/fimmu.2018.02936, PMID: 30619296 PMC6304371

[25] Subiza JL Palomares O Quinti I Sánchez-Ramón S. Editorial: trained immunity-based vaccines. Front In Immunol. (2021) 12:716296. doi: 10.3389/fimmu.2021.716296, PMID: 34249020 PMC8264451

[26] Ovsyannikova IG Reid KC Jacobson RM Oberg AL Klee GG Poland GA. Cytokine production patterns and antibody response to measles vaccine. Vaccine. (2003) 21:3946–53. doi: 10.1016/S0264-410X(03)00272-X, PMID: 12922130

[27] Kleinnijenhuis J Quintin J Preijers F Joosten LAB Ifrim DC Saeed S. Bacille Calmette-Guerin induces NOD2-dependent nonspecific protection from reinfection via epigenetic reprogramming of monocytes. Proc Natl Acad Sci United States America. (2012) 109:17537–42. doi: 10.1073/pnas.1202870109, PMID: 22988082 PMC3491454

[28] Kleinnijenhuis J Quintin J Preijers F Joosten LAB Jacobs C Xavier RJ. BCG-induced trained immunity in NK cells: Role for non-specific protection to infection. Clin Immunol (Orlando Fla.). (2014) 155:213–9. doi: 10.1016/j.clim.2014.10.005, PMID: 25451159 PMC5084088

[29] Kaufmann E Sanz J Dunn JL Khan N Mendonça LE Pacis A. BCG educates hematopoietic stem cells to generate protective innate immunity against tuberculosis. Cell. (2018) 172:176–190. doi: 10.1016/j.cell.2017.12.031, PMID: 29328912

[30] Ciarlo E Heinonen T Théroude C Asgari F Le Roy D Netea MG. Trained immunity confers broad-spectrum protection against bacterial infections. J Infect Dis. (2020) 222:1869–81. doi: 10.1093/infdis/jiz692, PMID: 31889191 PMC7653089

[31] Arunachalam PS Scott MKD Hagan T Li C Feng Y Wimmers F. Systems vaccinology of the BNT162b2 mRNA vaccine in humans. Nature. (2021) 596:410–6. doi: 10.1038/s41586-021-03791-x, PMID: 34252919 PMC8761119

[32] Debisarun PA Gössling KL Bulut O Kilic G Zoodsma M Liu Z. Induction of trained immunity by influenza vaccination - impact on COVID-19. PloS Pathog. (2021) 17:e1009928. doi: 10.1371/journal.ppat.1009928, PMID: 34695164 PMC8568262

[33] Wimmers F Donato M Kuo A Ashuach T Gupta S Li C. The single-cell epigenomic and transcriptional landscape of immunity to influenza vaccination. Cell. (2021) 184:3915–35. doi: 10.1016/j.cell.2021.05.039, PMID: 34174187 PMC8316438

[34] Brandi P Conejero L Cueto FJ Martínez-Cano S Dunphy G Gómez MJ. Trained immunity induction by the inactivated mucosal vaccine MV130 protects against experimental viral respiratory infections. Cell Rep. (2022) 38:110184. doi: 10.1016/j.celrep.2021.110184, PMID: 34986349 PMC8755442

[35] de Bree LCJ Koeken VACM Joosten LAB Aaby P Benn CS van Crevel R. Non-specific effects of vaccines: Current evidence and potential implications. Semin In Immunol. (2018) 39:35–43. doi: 10.1016/j.smim.2018.06.002, PMID: 30007489

[36] Balz K Trassl L Härtel V Nelson PP Skevaki C. Virus-induced T cell-mediated heterologous immunity and vaccine development. Front In Immunol. (2020) 11:513. doi: 10.3389/fimmu.2020.00513, PMID: 32296430 PMC7137989

[37] Yan J Nielsen TB Lu P Talyansky Y Slarve M Reza H. A protein-free vaccine stimulates innate immunity and protects against nosocomial pathogens. Sci Trans Med. (2023) 15:eadf9556. doi: 10.1126/scitranslmed.adf9556, PMID: 37792959 PMC10947341

[38] Cui X Shi Y Gu S Yan X Chen H Ge J. Antibacterial and antibiofilm activity of lactic acid bacteria isolated from traditional artisanal milk cheese from northeast China against enteropathogenic bacteria. Probiotics Antimicrobial Proteins. (2018) 10:601–10. doi: 10.1007/s12602-017-9364-9, PMID: 29218505

[39] Shi Z Guan N Sun W Sun T Niu L Li J. Protective Effect of Levilactobacillus brevis Against Yersinia enterocolitica Infection in Mouse Model via Regulating MAPK and NF-κB Pathway. Probiotics Antimicrobial Proteins. (2022) 14:830–44. doi: 10.1007/s12602-022-09957-x, PMID: 35665480

[40] Yang R Zhang S Yu Y Hong X Wang D Jiang Y. Adjuvant effects of bacterium-like particles in the intranasal vaccination of chickens against Newcastle disease. Veterinary Microbiol. (2021) 259:109144. doi: 10.1016/j.vetmic.2021.109144, PMID: 34111627

[41] Jia Z Niu L Guo J Wang J Li H Liu R. Pathogen-derived peptidoglycan skeleton enhances innate immune defense against Staphylococcus aureus via mTOR-HIF-1α-HK2-mediated trained immunity. Microbiological Res. (2025) 296:128160. doi: 10.1016/j.micres.2025.128160, PMID: 40174361

[42] Ferreira AV Kostidis S Groh LA Koeken VACM Bruno M Baydemir I. Dimethyl itaconate induces long-term innate immune responses and confers protection against infection. Cell Rep. (2023) 42:112658. doi: 10.1016/j.celrep.2023.112658, PMID: 37330914

[43] Paris S Chapat L Pasin M Lambiel M Sharrock TE Shukla R. β-glucan-induced trained immunity in dogs. Front In Immunol. (2020) 11:566893. doi: 10.3389/fimmu.2020.566893, PMID: 33162983 PMC7581789

[44] Pan Y Li J Xia X Wang J Jiang Q Yang J. β-glucan-coupled superparamagnetic iron oxide nanoparticles induce trained immunity to protect mice against sepsis. Theranostics. (2022) 12:675–88. doi: 10.7150/thno.64874, PMID: 34976207 PMC8692910

[45] Pasquali C Salami O Taneja M Gollwitzer ES Trompette A Pattaroni C. Enhanced mucosal antibody production and protection against respiratory infections following an orally administered bacterial extract. Front In Med. (2014) 1:41. doi: 10.3389/fmed.2014.00041, PMID: 25593914 PMC4292070

[46] Kim D Langmead B Salzberg SL. HISAT: a fast spliced aligner with low memory requirements. Nat Methods. (2015) 12:357–60. doi: 10.1038/nmeth.3317, PMID: 25751142 PMC4655817

[47] Anders S Pyl P T Huber W. HTSeq–A Python framework to work with high-throughput sequencing data. Bioinformatics. (2014), btu638, PMID: 25260700 10.1093/bioinformatics/btu638 PMC4287950

[48] Love MI Huber W Anders S. Moderated estimation of fold change and dispersion for RNA-seq data with DESeq2. Genome Biol. (2014) 15:550. doi: 10.1186/s13059-014-0550-8, PMID: 25516281 PMC4302049

[49] Subramanian A Tamayo P Mootha VK Mukherjee S Ebert BL Gillette MA. Gene set enrichment analysis: a knowledge-based approach for interpreting genome-wide expression profiles. Proc Natl Acad Sci United States America. (2005) 102:15545–50. doi: 10.1073/pnas.0506580102, PMID: 16199517 PMC1239896

[50] Draghici S Khatri P Tarca AL Amin K Done A Voichita C. A systems biology approach for pathway level analysis. Genome Res. (2007) 17:1537–45. doi: 10.1101/gr.6202607, PMID: 17785539 PMC1987343

[51] Cheng SX Lightfoot YL Yang T Zadeh M Tang L Sahay B. Epithelial CaSR deficiency alters intestinal integrity and promotes proinflammatory immune responses. FEBS Lett. (2014) 588:4158–66. doi: 10.1016/j.febslet.2014.05.007, PMID: 24842610 PMC4234694

[52] Chen S-N Tan Y Xiao X-C Li Q Wu Q Peng Y-Y. Deletion of TLR4 attenuates lipopolysaccharide-induced acute liver injury by inhibiting inflammation and apoptosis. Acta Pharmacologica Sin. (2021) 42:1610–9. doi: 10.1038/s41401-020-00597-x, PMID: 33495514 PMC8463538

[53] Gu H Zeng X Peng L Xiang C Zhou Y Zhang X. Vaccination induces rapid protection against bacterial pneumonia via training alveolar macrophage in mice. ELife. (2021) 10:e69951. doi: 10.7554/eLife.69951, PMID: 34544549 PMC8455131

[54] Nasiri-Ansari N Nikolopoulou C Papoutsi K Kyrou I Mantzoros CS Kyriakopoulos G. Empagliflozin attenuates non-alcoholic fatty liver disease (NAFLD) in high fat diet fed apoE(-/-) mice by activating autophagy and reducing ER stress and apoptosis. Int J Mol Sci. (2021) 22:818. doi: 10.3390/ijms22020818, PMID: 33467546 PMC7829901

[55] Ye J Zhong S Deng Y Yao X Liu Q Wang J-Z. HDAC7 activates IKK/NF-κB signaling to regulate astrocyte-mediated inflammation. Mol Neurobiol. (2022) 59:6141–57. doi: 10.1007/s12035-022-02965-6, PMID: 35871708 PMC9309093

[56] Zhong W-J Liu T Yang H-H Duan J-X Yang J-T Guan X-X. TREM-1 governs NLRP3 inflammasome activation of macrophages by firing up glycolysis in acute lung injury. Int J Biol Sci. (2023) 19:242–57. doi: 10.7150/ijbs.77304, PMID: 36594089 PMC9760435

[57] Livak KJ Schmittgen TD. Analysis of relative gene expression data using real-time quantitative PCR and the 2(-Delta Delta C(T)) Method. Methods (San Diego Calif.). (2001) 25:402–8., PMID: 11846609 10.1006/meth.2001.1262

[58] Nour MA El-Hindawy MM Abou-Kassem DE Ashour EA Abd El-Hack ME Mahgoub S. Productive performance, fertility and hatchability, blood indices and gut microbial load in laying quails as affected by two types of probiotic bacteria. Saudi J Biol Sci. (2021) 28:6544–55. doi: 10.1016/j.sjbs.2021.07.030, PMID: 34764770 PMC8568992

[59] McCrudden CM Bennie L Chambers P Wilson J Kerr M Ziminska M. Peptide delivery of a multivalent mRNA SARS-CoV-2 vaccine. J Controlled Release: Off J Controlled Release Soc. (2023) 362:536–47. doi: 10.1016/j.jconrel.2023.08.053, PMID: 37648082

[60] Cheng S-C Quintin J Cramer RA Shepardson KM Saeed S Kumar V. mTOR- and HIF-1α-mediated aerobic glycolysis as metabolic basis for trained immunity. Sci (New York N.Y.). (2014) 345:1250684. doi: 10.1126/science.1250684, PMID: 25258083 PMC4226238

[61] Roewe J Stavrides G Strueve M Sharma A Marini F Mann A. Bacterial polyphosphates interfere with the innate host defense to infection. Nat Commun. (2020) 11:4035. doi: 10.1038/s41467-020-17639-x, PMID: 32788578 PMC7423913

[62] Lowy FD. Antimicrobial resistance: the example of Staphylococcus aureus. J Clin Invest. (2003) 111:1265–73. doi: 10.1172/JCI18535, PMID: 12727914 PMC154455

[63] Wowk PF Franco LH Fonseca DMD Paula MO Vianna É.D.S.O. Wendling AP. Mycobacterial Hsp65 antigen upregulates the cellular immune response of healthy individuals compared with tuberculosis patients. Hum Vaccines Immunotherapeutics. (2017) 13:1040–50. doi: 10.1080/21645515.2016.1264547, PMID: 28059670 PMC5443371

[64] Qiao Y Zhang Y Chen J Jin S Shan Y. A biepitope, adjuvant-free, self-assembled influenza nanovaccine provides cross-protection against H3N2 and H1N1 viruses in mice. Nano Res. (2022) 15:8304–14. doi: 10.1007/s12274-022-4482-4, PMID: 35911479 PMC9325945

[65] van der Meer JWM Joosten LAB Riksen N Netea MG. Trained immunity: A smart way to enhance innate immune defence. Mol Immunol. (2015) 68:40–4. doi: 10.1016/j.molimm.2015.06.019, PMID: 26597205

[66] Waikhom D Kezhedath J Krishnan R Varghese T Kurcheti PP Valappil RK. Beta-glucan stimulation induces trained immunity markers in common carp, Cyprinus carpio. Fish Shellfish Immunol. (2022) 131:855–61. doi: 10.1016/j.fsi.2022.10.069, PMID: 36336239

[67] Jeyanathan M Vaseghi-Shanjani M Afkhami S Grondin JA Kang A D'Agostino MR. Parenteral BCG vaccine induces lung-resident memory macrophages and trained immunity via the gut-lung axis. Nat Immunol. (2022) 23:1687–702. doi: 10.1038/s41590-022-01354-4, PMID: 36456739 PMC9747617

[68] Suzuki I Tanaka H Kinoshita A Oikawa S Osawa M Yadomae T. Effect of orally administered beta-glucan on macrophage function in mice. Int J Immunopharmacol. (1990) 12:675–84. doi: 10.1016/0192-0561(90)90105-V, PMID: 2272730

[69] Netea MG Joosten LAB. Trained immunity and local innate immune memory in the lung. Cell. (2018) 175:1463–5. doi: 10.1016/j.cell.2018.11.007, PMID: 30500533

[70] Liang J Zhu F Cheng K Ma N Ma X Feng Q. Outer membrane vesicle-based nanohybrids target tumor-associated macrophages to enhance trained immunity-related vaccine-generated antitumor activity. Advanced Materials (Deerfield Beach Fla.). (2023) 35:e2306158. doi: 10.1002/adma.202306158, PMID: 37643537

[71] Pellon A Barriales D Peña-Cearra A Castelo-Careaga J Palacios A Lopez N. The commensal bacterium Lactiplantibacillus plantarum imprints innate memory-like responses in mononuclear phagocytes. Gut Microbes. (2021) 13:1939598. doi: 10.1080/19490976.2021.1939598, PMID: 34224309 PMC8259724

[72] Tosi MF. Innate immune responses to infection. J Allergy Clin Immunol. (2005) 116:241–49. doi: 10.1016/j.jaci.2005.05.036, PMID: 16083775

[73] Keijzer C Haijema BJ Meijerhof T Voorn P de Haan A Leenhouts K. Inactivated influenza vaccine adjuvanted with bacterium-like particles induce systemic and mucosal influenza A virus specific T-cell and B-cell responses after nasal administration in a TLR2 dependent fashion. Vaccine. (2014) 32:2904–10. doi: 10.1016/j.vaccine.2014.02.019, PMID: 24598720

[74] Wang J Lan Q Zong X Zhu G Yang R Yang G. Protection against genotype VII Newcastle disease virus by a mucosal subunit vaccination based on bacterium-like particles bearing the F or HN antigen. Int J Biol Macromolecules. (2023) 244:125293. doi: 10.1016/j.ijbiomac.2023.125293, PMID: 37315677

[75] Xu W Zhao M Fu X Hou J Wang Y Shi F. Molecular mechanisms underlying macrophage immunomodulatory activity of Rubus chingii Hu polysaccharides. Int J Biol Macromolecules. (2021) 185:907–16. doi: 10.1016/j.ijbiomac.2021.07.024, PMID: 34242647

[76] Charras A Arvaniti P Le Dantec C Arleevskaya MI Zachou K Dalekos GN. JAK inhibitors suppress innate epigenetic reprogramming: a promise for patients with sjögren's syndrome. Clin Rev In Allergy Immunol. (2020) 58:182–93. doi: 10.1007/s12016-019-08743-y, PMID: 31165348

[77] Xu Q Briggs J Park S Niu G Kortylewski M Zhang S. Targeting Stat3 blocks both HIF-1 and VEGF expression induced by multiple oncogenic growth signaling pathways. Oncogene. (2005) 24:5552–60. doi: 10.1038/sj.onc.1208719, PMID: 16007214

[78] Dimidi E Christodoulides S Scott SM Whelan K. Mechanisms of action of probiotics and the gastrointestinal microbiota on gut motility and constipation. Adv In Nutr (Bethesda Md.). (2017) 8:484–94. doi: 10.3945/an.116.014407, PMID: 28507013 PMC5421123

[79] Audouy SAL van Selm S van Roosmalen ML Post E Kanninga R Neef J. Development of lactococcal GEM-based pneumococcal vaccines. Vaccine. (2007) 25:2497–506. doi: 10.1016/j.vaccine.2006.09.026, PMID: 17081660

