# Supplementary material for: Probiotic peptidoglycan skeleton enhances vaccine efficacy against MRSA by inducing trained immunity via the TLR2/JAK-STAT3 pathway
Source: Front Immunol. 2025 Jul 18;16:1606626. doi: 10.3389/fimmu.2025.1606626 (PMC12314533; doi:10.3389/fimmu.2025.1606626)
Supplement: Supplementary file 1 [file DataSheet1.docx]

TableS1 Primer design

| Gene name | Sequence (5’-3’) | Length(bp) |
| --- | --- | --- |
| CCL2 | F: TTAAAAACCTGGATCGGAACCAA | 121 |
|  | R: GCATTAGCTTCAGATTTACGGGT |  |
| CCL7 | F: CCACCATGAGGATCTCTGC | 115 |
|  | R: TTGACATAGCAGCATGTGGAT |  |
| mTORC2 | F: CCATCCAATCTGATGCTGGA | 186 |
|  | R: GGTGTGGCATGTGGTTCTGT |  |
| HDAC7 | F: CGCAGCCAGTGTGAGTGTCT | 214 |
|  | R: GCTCGTTCCAGATGGTGTCAGTA |  |
| HIF1α | F: ACCTTCATCGGAAACTCCAAAG | 187 |
|  | R: ACTGTTAGGCTCAGGTGAACT |  |
| β-actin | F: CCAGTTGGTAACAATGCCATGT | 154 |
|  | R: GGCTGTATTCCCCTCCATCG |  |
| IL-1β | F: TTCAGGCAGGCAGTATCACTC | 75 |
|  | R: GAAGGTCCACGGGAAAGACAC |  |
| TNF-α | F: CCCCAAAGGGATGAGAAGTT | 132 |
|  | R: CACTTGGTGGTTTGCTACGA |  |
| IL-6 | F: AAAGAGTTGTGCAATGGCAATTCT | 51 |
|  | R: AAGTGCATCATCGTTCATACA |  |
| IFN-β | F: AGACAATCAGGCCATCAGCA | 135 |
|  | R: TGGACCTGTGGGTTGTTGAC |  |
| IL-10 | F: CGCAGCTCTAGGAGCATGTG | 105 |
|  | R:GCTCTTACTGACTGGCATGAG |  |
| IL-12 | F: ACAGCACCAGCTTCTTCATCAG | 75 |
|  | R:TCTTCAAAGGCTTCATCTGCAA |  |
| TLR2 | F: GGGGTGTGTGATGGCCGCTC | 137 |
|  | R: TGGAGGTTCGCACACGCTCG |  |
| TLR4 | F: ATGGCATGGCTTACACCACC | 129 |
|  | R: GAGGCCAATTTTGTCTCCACA |  |


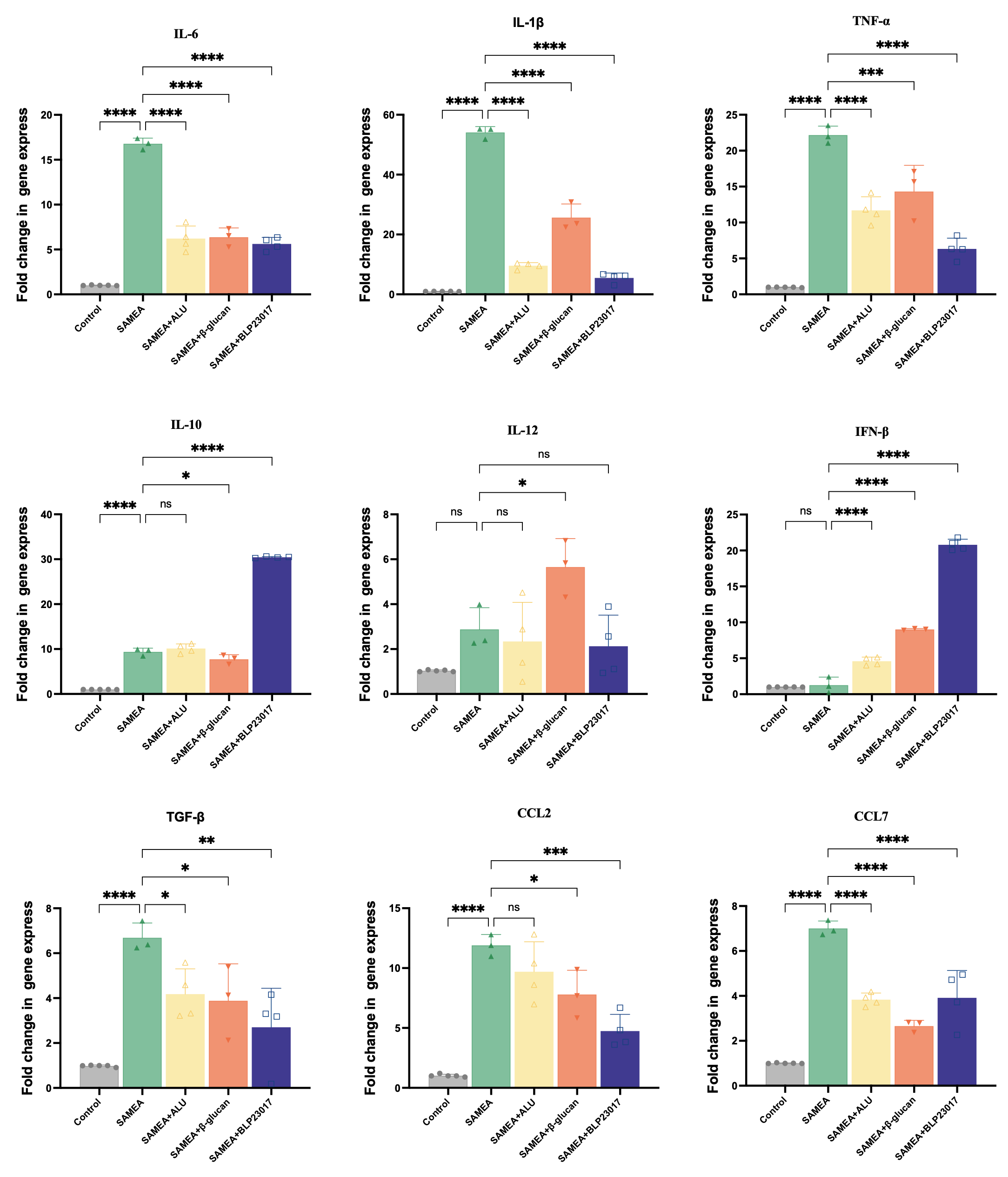


Figure S1. Cytokine expression after two-dose subunit vaccine immunization. The mRNA relative expression levels of IL-6, IL-1β, TNF-α, IL-10, IL-12, IFN-β, TGF-β, CCL2, and CCL7 on Day 63 in the kidney were measured by RT-qPCR and expressed using the 2^−ΔΔCT^ value. (* *p* < 0.05, ** *p* < 0.01, *** *p* < 0.001, **** *p* < 0.0001).


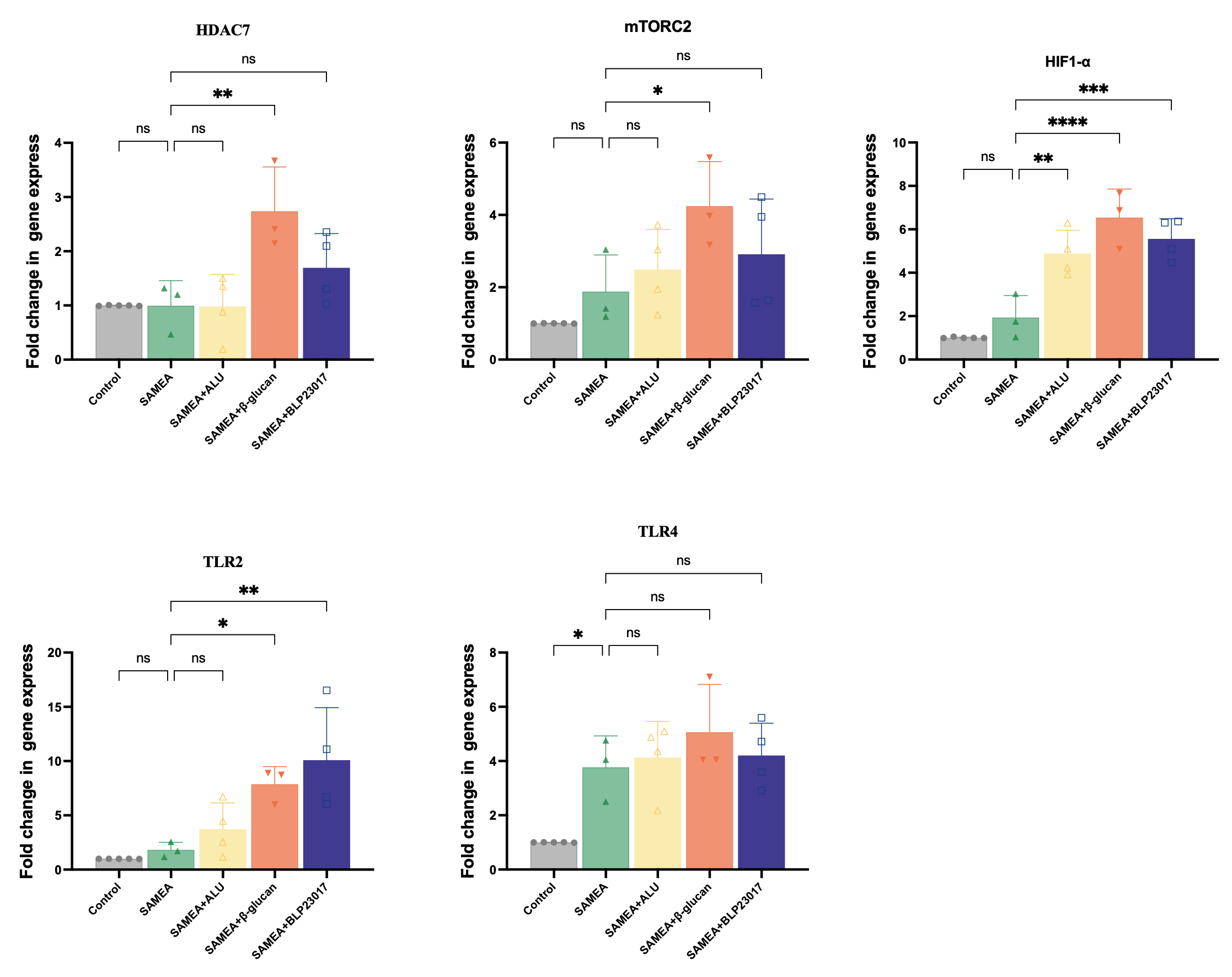


Figure S2. Cytokine expression after two-dose subunit vaccine immunization. The mRNA relative expression levels of TLR2, TLR4, HDAC7, mTORC2, and HIF1-α on Day 63 in the kidney were measured by RT-qPCR and expressed using the 2^−ΔΔCT^ value. (* *p* < 0.05, ** *p* < 0.01, *** *p* < 0.001, **** *p* < 0.0001).


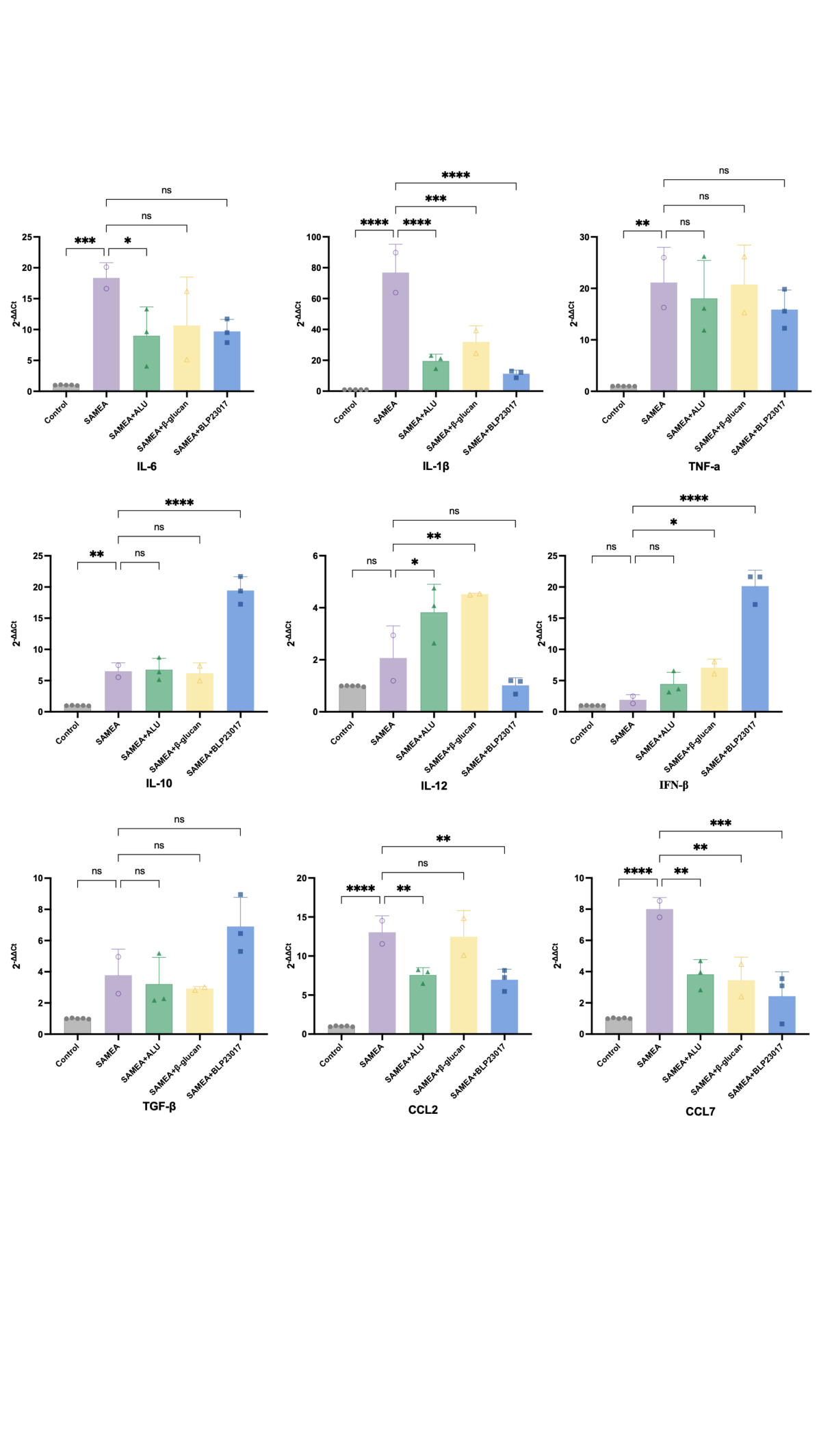


Figure S3. Cytokine expression after one-dose subunit vaccine immunization. Relative expression levels of cytokines on Day 42 of IL-6, IL-1β, TNF-α, IL-10, IL-12, IFN-β, TGF-β, CCL2, and CCL7 in the kidney were measured by RT-qPCR and expressed using the 2^−ΔΔCT^ value. (* *p* < 0.05, ** *p* < 0.01, *** *p* < 0.001, **** *p* < 0.0001).


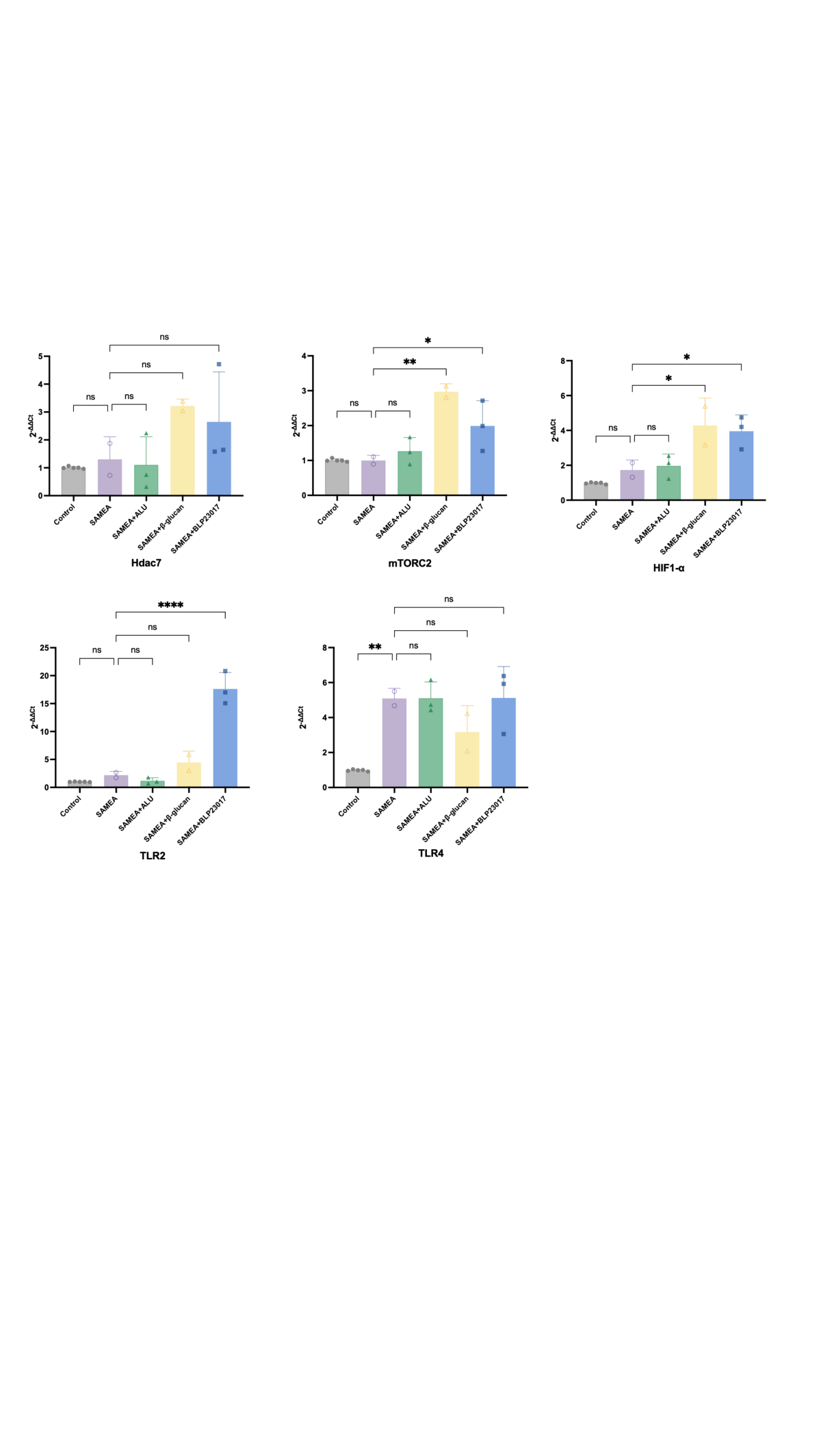


Figure S4. Cytokine expression after one-dose subunit vaccine immunization. Relative expression levels of cytokines on Day 42 of TLR2, TLR4, HDAC7, mTORC2, and HIF1-α in the kidney were measured by RT-qPCR and expressed using the 2^−ΔΔCT^ value. (* *p* < 0.05, ** *p* < 0.01, *** *p* < 0.001, **** *p* < 0.0001).


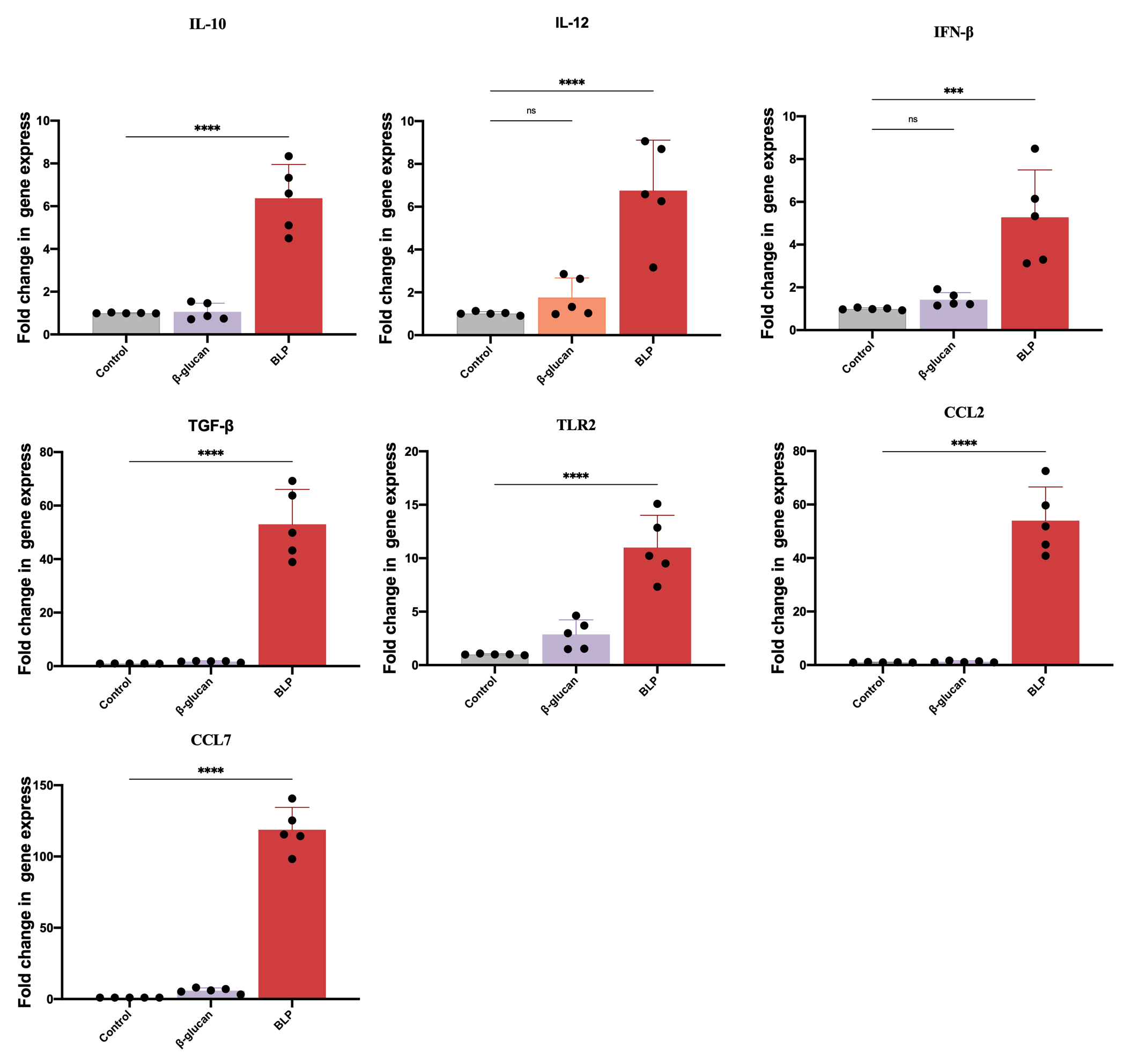


Figure S5. BLP induced trained immunity in Raw264.7 cells. The mRNA relative expression levels of IL-10, IL-12, IFN-β, TGF-β, TLR2, CCL2, and CCL7 on the D8. The results of qPCR were expressed using the 2^−ΔΔCT^ value. (* *p* < 0.05, ** *p* < 0.01, *** *p* < 0.001, **** *p* < 0.0001).


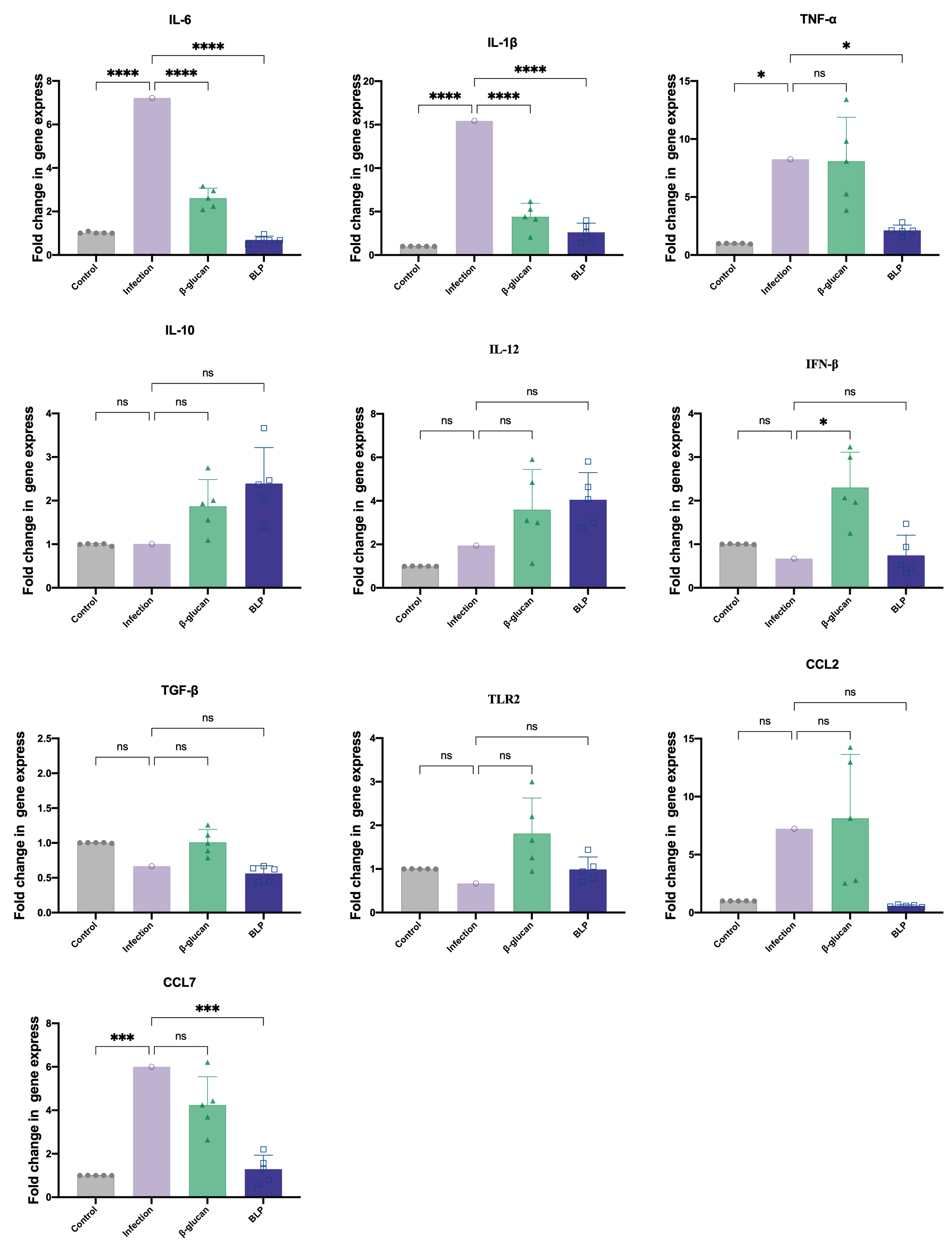


Figure S6. Trained immunity induced by BLP rapidly protected mice infected with *S. aureus*. The mRNA relative express levels of IL-6, IL-1β, TNF-α, IL-10, IL-12, IFN-β, TGF-β, TLR2, CCL2 and CCL7 on the D12 in spleen. The results of qPCR were expressed using the 2^−ΔΔCT^ value. (* *p* < 0.05, ** *p* < 0.01, *** *p* < 0.001, **** *p* < 0.0001).


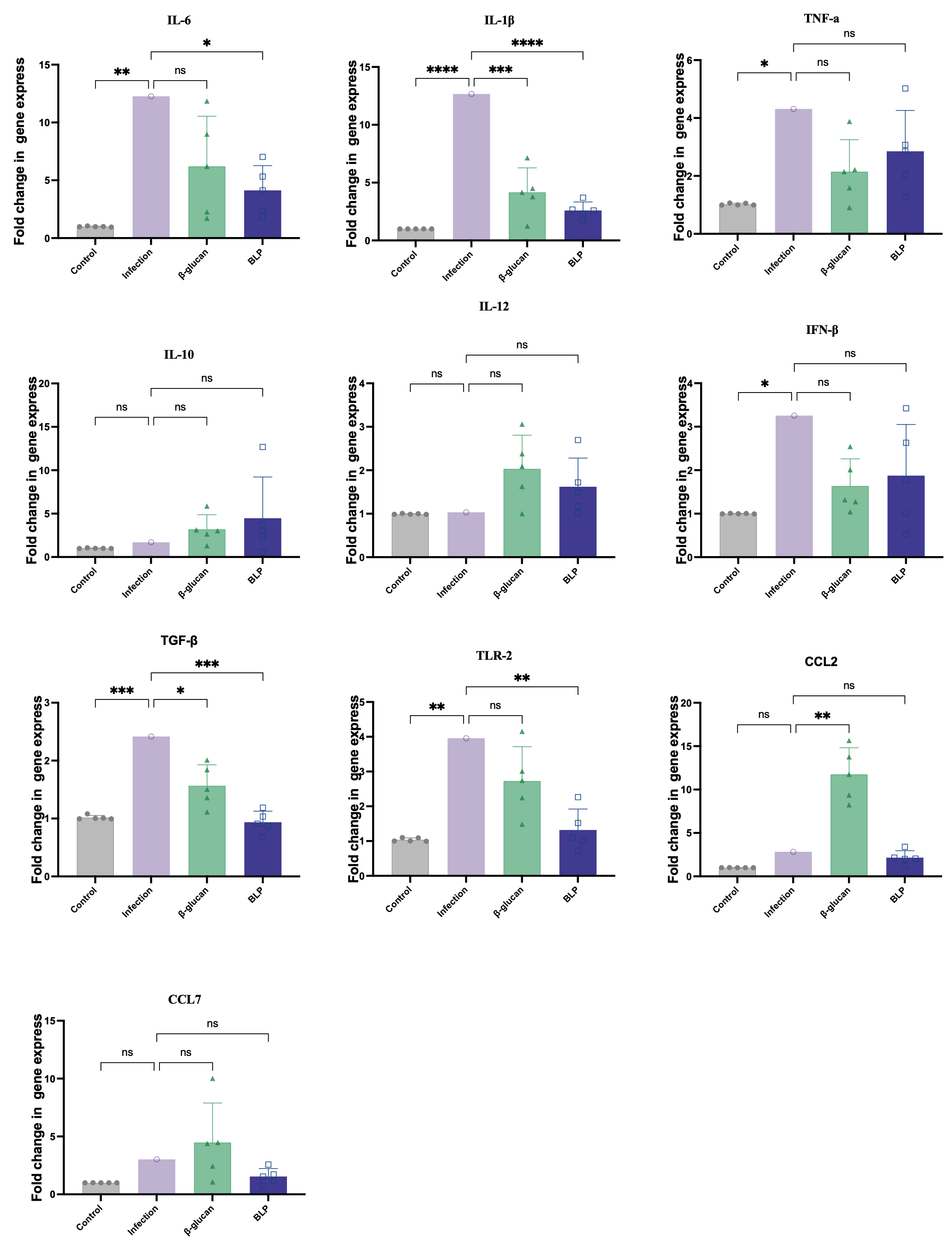


Figure S7. Trained immunity induced by BLP rapidly protected mice infected with *S. aureus*. The mRNA relative express levels of IL-6, IL-1β, TNF-α, IL-10, IL-12, IFN-β, TGF-β, TLR2, CCL2 and CCL7 on the D12 in kidney. The results of qPCR were expressed using the 2^−ΔΔCT^ value. (* *p* < 0.05, ** *p* < 0.01, *** *p* < 0.001, **** *p* < 0.0001).


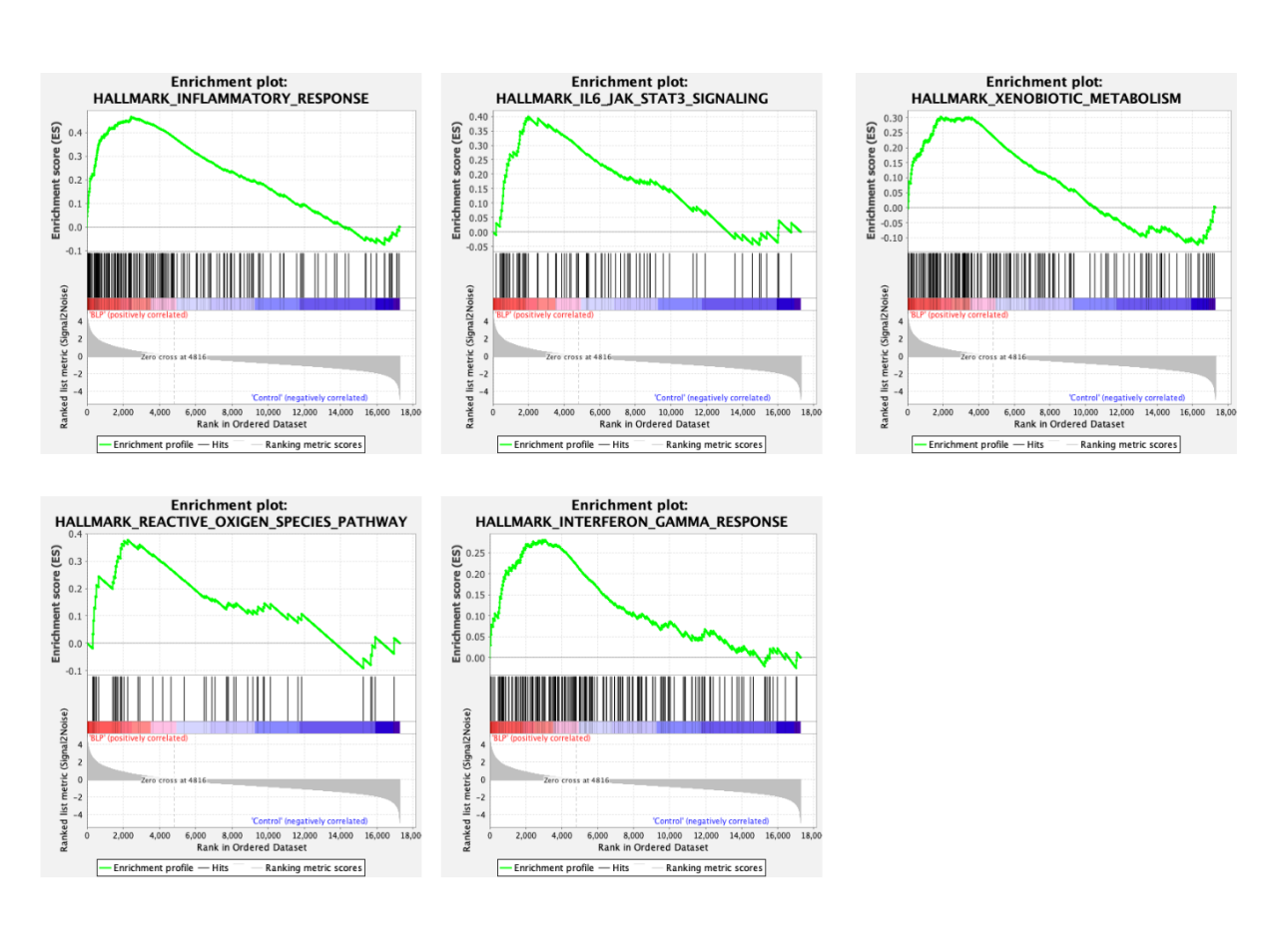


Figure S8. Gene set enrichment analysis (GSEA) using the Molecular Signatures Database (MSigDB) Hallmark gene set collection. Differentially expressed gene sets in BLP-treated monocytes versus controls.
